# A new era in neuropharmacology: assessing the efficacy and safety of novel anti-amyloid and non-amyloid drug targets for Alzheimer's disease

**DOI:** 10.1007/s00415-026-13829-7

**Published:** 2026-05-05

**Authors:** Mohamed M. Hafez, Haidy A. Abbas, Nabil A. Shoman, Ayman A. Soubh, Omnia Aly, Mohamed F. Sallam, Mahmoud Seliem, Fady A. Malaak

**Affiliations:** 1 Department of Biochemistry, Faculty of Pharmacy, Ahram Canadian University, Giza, Egypt; 2 Department of Pharmacognosy, Faculty of Pharmacy, Ahram Canadian University, Giza, Egypt; 3 Department of Pharmaceutics and Pharmaceutical Technology, Faculty of Pharmacy, Ahram Canadian University, Giza, Egypt; 4 Department of Pharmacology and Toxicology, School of Life and Medical Sciences, University of Hertfordshire Hosted By Global Academic Foundation, New Administrative Capital, Cairo, Egypt; 5 Department of Medical Biochemistry, National Research Centre, Cairo, Egypt; 6 Department of Pharmacology and Toxicology, Faculty of Pharmacy, Ahram Canadian University, Giza, Egypt

**Keywords:** Alzheimer’s disease, Anti-amyloid monoclonal antibodies, Neuroinflammation, Biomarkers, Tau-centric therapies, Precision neuropharmacology

## Abstract

**Background:**

Alzheimer disease (AD) is the most common cause of dementia in the world with the prevalence expected to increase threefold to 152.8 million people by 2050. The current medications provide a short-term ameliorative effect, and this requires development of disease-modifying treatments, which address the biological pathogenesis.

**Methods:**

This review assesses the changing neuropharmacological environment offering a critical analysis of anti-amyloid monoclonal antibodies and investigates the so-called expanding frontier of non-amyloid targets. It also examines the approaches of clinical trials and the trend of biomarker-based patient selection and precision medicine.

**Results:**

Although β-site APP-cleaving enzyme 1 (BACE1) and secretase inhibitors did not achieve success in clinical trials because of mechanism-based toxicity and cognitive impairment, new monoclonal antibodies such as lecanemab and donanemab have shown high amyloid plaque clearance and reduced cognitive deterioration. Nevertheless, the treatments are associated with amyloid-related imaging abnormalities (ARIA). In addition to amyloid, studies are focusing on tau hyperphosphorylation, neuroinflammation through triggering receptor on myeloid cells 2 (TREM2) and NLR family pyrin domain containing 3 (NLRP3) and growth factor-mediated synaptic plasticity through brain-derived neurotrophic factor (BDNF).

**Conclusions:**

AD treatment has entered the new era that demands a paradigm shift from monotherapies to multi-target cocktails. The future lies in precision neuropharmacology, where genetic stratification and individual biomarker analysis are used to provide the correct treatment at the most appropriate biological stage.

## Introduction

Alzheimer’s disease (AD) represents a formidable challenge to modern healthcare, standing as the leading cause of dementia globally [16]. Driven by an aging population, the prevalence of AD is projected to triple by 2050, reaching approximately 152.8 million individuals [36]. This demographic shift is expected to exert unprecedented pressure on health services and economies, with global costs projected to reach trillions of dollars in the coming years. Consequently, there is an urgent imperative to develop interventions that extend beyond symptomatic relief to delay the onset or slow disease progression [12, 15].

Existing medications, including acetylcholinesterase inhibitors and memantine, provide only temporary relief by modulating neurotransmitter levels to alleviate symptoms, without effecting any substantial change in the disease’s gradual progression [94, 126]. This glaring discrepancy between the extent of human suffering and the limited nature of available treatments highlights a critical and urgent clinical need. Despite years of dedicated research, the treatment landscape has long been marked by a substantial divide between our understanding of the disease’s complexities and our capacity to halt its progression. This divide has prompted a reassessment of our drug discovery approach, shifting from mere symptom management to more intricate biological interventions that address the underlying causes of neuronal death [36].

The pathophysiology of AD, which involves proteins and biological pathways other than amyloid-beta (A*β*), is still poorly understood [69]. The great biological complexity of the disease is intrinsically connected to the struggle to create effective medicines. The classic "amyloid cascade hypothesis," which proposed A*β* accumulation as the primary cause of the disease, has been essential [64]. Even while A*β* accumulation is a crucial feature, the pathophysiology of AD is now recognized as a complicated cascade. This disease, which is more strongly linked to neuronal loss and cognitive decline, comprises persistent neuroinflammation, synaptic dysfunction, vascular dysregulation, and the ensuing hyperphosphorylation and aggregation of tau protein into neurofibrillary tangles [95]. At the molecular scale, AD involves the accumulation of A*β* plaques alongside the formation of abnormally phosphorylated tau protein tangles inside neurons. These changes disrupt synaptic communication, promote neurodegeneration, and trigger inflammatory responses in neural tissue. Recent critical reviews have challenged the classical amyloid cascade model, showing that the link between A*β* deposits and how severe the disease becomes may not be as strong as scientists once believed. This suggests that other disease processes occurring later might actually be more important in advancing Alzheimer’s progression [97].

Oxidative stress is another major contributor to the disease. It happens when there is too much production of harmful reactive oxygen and nitrogen molecules compared to the brain’s own protective antioxidant mechanisms. This creates damaging oxidative conditions that make neuronal problems worse [97, 133].

Additionally, neuroinflammation also plays a critical role, where immune cells in the brain, microglia, and astrocytes become overactive. This overactivity triggers the release of inflammatory molecules that damage synaptic connections and promote neurodegeneration [18, 69]. Emerging evidence also suggests that systemic inflammation and immune dysregulation contribute to AD onset and progression, with "inflammaging", the age-related increase in pro-inflammatory mediators, potentially amplifying neuroinflammation [13]. Because all these different disease mechanisms interact in such complicated ways, targeting just one pathway will not be enough to treat Alzheimer’s effectively. This creates a huge challenge for developing treatments and shows why we need strategies that attack disease from multiple angles at once.

This urgent need has pushed researchers to rethink their approach to Alzheimer’s drug development. Instead of focusing almost exclusively on A*β*, scientists are now exploring a much broader range of therapeutic targets that better reflect how complex the disease really is [140]. After years of disappointing results, the field has reached a pivotal inflection point. For a long time, treatments only aimed to ease symptoms temporarily, but now the goal has shifted toward changing how the disease progresses. Today’s research strategies recognize that Alzheimer’s does not have a single cause, so scientists are investigating multiple pathways beyond amyloid that contribute to nerve cell death, brain inflammation, and the breakdown of connections between neurons [36, 37]. This shift in thinking has changed the drug development landscape significantly. About 70% of Alzheimer’s medications currently being tested target these newer pathways rather than just amyloid [36]. This new era in neuropharmacology emphasizes the exploration of novel anti-amyloid and non-amyloid drug targets to achieve a more profound and lasting therapeutic impact. It is an era defined by the rigorous clinical evaluation of novel agents, most notably monoclonal antibodies like lecanemab [136] and donanemab [129]. These medications have shown, for the first time, that removing amyloid plaques from the brain can slow down cognitive decline in people with early Alzheimer’s. The treatment effects are statistically significant, though clinical magnitude remains a subject of discussion [82, 129, 136]. In fact, lecanemab and aducanumab have been approved in the USA for treating early-stage Alzheimer’s, making them the first treatments that actually modify the disease process rather than just temporarily relieving symptoms [33, 34]. These antibody drugs work by substantially lowering A*β* levels in the brain, and studies show they can slow cognitive deterioration when they successfully reduce measurable plaque A*β* accumulation [34].

Beyond amyloid, researchers are now actively investigating a wide range of other targets, including tau protein, brain inflammation, and ways to strengthen synaptic connections. This reflects a more complete picture of what is actually happening in Alzheimer’s disease [35, 36].

This review will evaluate the current landscape of Alzheimer’s disease therapeutics. It provides a critical appraisal of anti-amyloid monoclonal antibodies, analyzing their efficacy and safety, before exploring the expanding frontier of non-amyloid targets. The discussion will address modern clinical trial design and biomarker use, concluding that the future of effective treatment lies in precision medicine and multi-target strategies designed to meaningfully alter the disease course.

## Targeting the amyloid cascade: the traditional therapeutic paradigm in Alzheimer’s disease

### Rationale for amyloid-centered therapeutic intervention

**Fig. 1:**
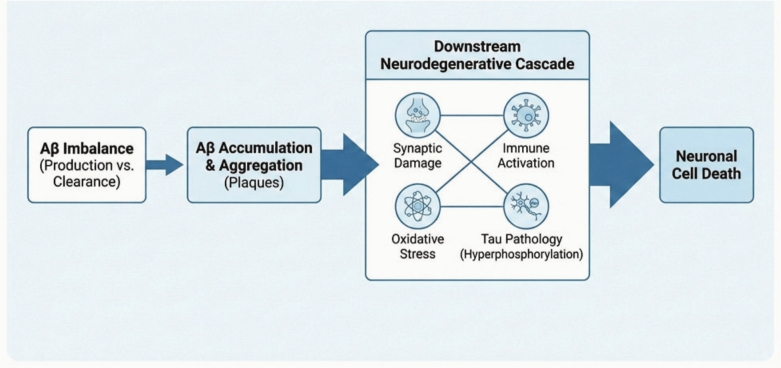
The traditional amyloid cascade hypothesis

The amyloid cascade hypothesis serves as the primary theoretical framework which scientists use to study AD development after its discovery in the 1980 s [80]. This hypothesis proposes that an imbalance between the production and clearance of Aβ peptides leads to their abnormal accumulation and aggregation within the brain parenchyma. The disease process starts when pathological substances build up in the body which trigger a complex neurodegenerative process that damages synapses and triggers immune system activation and produces oxidative stress and tau protein hyperphosphorylation before it causes neuronal cell death [5] **(**Fig. 1**).**

Significantly, early confirmation of amyloid deposition in vivo is provided by cerebrospinal fluid biomarkers and amyloid positron emission tomography imaging many years prior to overt cognitive symptoms. This separation in time of pathology from cognitive decline has strengthened the case for amyloid-based therapeutic intervention, especially early in the disease. As a result, although its primacy continues to be disputed, the amyloid cascade hypothesis remains pivotal for the development of disease-modifying treatments that target the natural history of AD as opposed to only providing symptomatic benefit [77].

### Upstream intervention: inhibition of Aβ production

A critical distinction is now clearly recognized between early-onset familial AD and late-onset sporadic AD. Familial AD is largely driven by genetic mutations (in amyloid precursor protein (APP), PSEN1, or PSEN2) that increase A*β* production [120]. In contrast, sporadic AD increasingly appears to reflect a failure of proteostasis networks, where clearance mechanisms cannot keep up with ongoing A*β* generation [98]. This conceptual shift has reshaped therapeutic strategies, splitting amyloid-targeted approaches into two main paths: preventing new A*β* from being made (upstream inhibition) or improving the removal of pathogenic species that have already formed (downstream clearance) [61].

The production of A*β* is governed by the sequential proteolytic cleavage of the transmembrane APP. This process involves two key enzymes: β-site APP-cleaving enzyme 1 (BACE1) and the γ-secretase complex. Theoretically, inhibiting these enzymes offers the most direct method to halt the amyloid load at its source [139]. However, the translation of this theory into clinical practice has been fraught with failure, revealing the intricate physiological roles these enzymes play beyond amyloid generation. The saga of secretase inhibition serves as a cautionary tale in drug development, highlighting the dangers of targeting enzymes with broad substrate profiles.

#### β-Secretase (BACE1) inhibition

β-Site APP-cleaving enzyme 1 (BACE1) activates the amyloidogenic pathway by cleaving APP at the Asp + 1 site, releasing soluble APPβ and a membrane-bound C99 fragment [85]. Because this cleavage is the rate-limiting step in the production of Aβ, BACE1 has traditionally been considered an ideal pharmacological target. Potent, brain-penetrant, inhibitors like verubecestat, lanabecestat, and elenbecestat were able to reduce cerebrospinal fluid Aβ levels by 80–90% in Phase II and III clinical trials [141]. However, the "BACE inhibitor paradox" refers to the finding that while these drugs successfully engaged their target and lowered amyloid markers, they consistently failed to improve cognition and, in many cases, worsened it.

#### The mechanism of clinical failure: cognitive worsening

Despite effective target engagement, BACE1 inhibitors such as verubecestat, lanabecestat, atabecestat, and umibecestat uniformly failed in Phase II and III clinical trials. Importantly, these trials were not merely futile; in many cases, they were detrimental. A consistent pattern emerged in which patients receiving BACE1 inhibitors exhibited dose-dependent cognitive worsening compared with placebo [141]. This decline was frequently detectable within months of treatment initiation, strongly suggesting a synaptic or functional deficit rather than progressive structural neurodegeneration.

Subsequent mechanistic investigations revealed that these adverse outcomes arise from the broad physiological roles of BACE1, which extend far beyond APP processing. BACE1 is now recognized as a promiscuous protease essential for the cleavage of multiple substrates critical for neuronal development, synaptic plasticity, and circuit integrity. Among the most relevant non-APP substrates are seizure protein 6 (SEZ6), close homolog of L1 (CHL1), and neuregulin-1 (NRG1) [111].

Seizure protein 6 (SEZ6) is a transmembrane protein that plays an essential role in dendritic spine formation, synaptic maintenance, and activity-dependent plasticity and utilizes BACE1 as its exclusive sheddase. Consequently, the pharmacological inhibition of BACE1 leads to a marked reduction of soluble SEZ6 levels in the cerebrospinal fluid. Both preclinical and clinical studies have demonstrated that this diminished SEZ6 shedding is associated with reduced dendritic spine density in hippocampal neurons, resulting in impaired synaptic plasticity and memory formation [121]. Accordingly, this mechanism is now widely regarded as a primary driver of the synaptic toxicity and rapid cognitive decline observed in BACE1 inhibitor trials.

Close homolog of L1 (CHL1) is a neural cell adhesion molecule that relies on BACE1-mediated cleavage to ensure proper axonal guidance and the organization of neural circuits. When this processing is disrupted, it has been linked to axonal misrouting and structural disorganization, specifically within hippocampal mossy fiber pathways. These structural alterations offer a compelling biological explanation for observed cognitive deficits, distinct from amyloid-related mechanisms [112].

Neuregulin-1 (NRG1) relies on BACE1 cleavage for normal myelination during development and the maintenance of muscle spindle function in adulthood. While hypomyelination was primarily a concern derived from BACE1 knockout models, pharmacological inhibition has been specifically shown to disrupt muscle spindle integrity and proprioceptive signaling. The accumulation of full-length NRG1 due to impaired cleavage alters downstream signaling pathways, potentially contributing to the motor, psychiatric, and coordination-related adverse effects reported in high-dose clinical cohorts [68].

Collectively, these findings indicate that excessive inhibition of BACE1 induces mechanism-based synaptic toxicity that outweighs the potential benefits of amyloid reduction. Consequently, long-term, high-dose BACE1 inhibition in symptomatic Alzheimer’s disease patients is no longer considered a viable therapeutic strategy [30]. Current research efforts have therefore shifted toward sub-maximal inhibition or intervention at pre-symptomatic stages, approaches that remain biologically plausible but have yet to demonstrate clinical efficacy.

#### γ-Secretase inhibition and modulation

After cleavage of APP by BACE1, the last intramembrane cleavage of C99 is performed by the γ-secretase complex of the four proteins, including presenilin, nicastrin, APH-1, and PEN-2, to release Aβ-peptides of different lengths [93]. First-generation γ-secretase inhibitors such as semagacestat were able to reduce Aβ production, but were eventually unsuccessful in the clinic due to severe toxicities. These undesirable outcomes were the result of non-selective inhibition of the Notch signaling, which is a pathway vital to cell differentiation and immune homeostasis and also depends on γ-secretase [132].

In response, the field shifted toward second-generation γ-secretase modulators (GSMs). Unlike γ-secretase inhibitors, GSMs bind allosterically to the enzyme complex, inducing conformational changes that shift cleavage away from longer, more toxic Aβ species, particularly Aβ₄₂, toward shorter, less amyloidogenic peptides such as Aβ₃₇ and Aβ₃₈, while preserving overall enzymatic activity and Notch signaling [50]. Although GSMs exhibit improved safety profiles in preclinical models, their clinical efficacy has remained modest, likely reflecting their inability to remove pre-existing amyloid deposits or reverse downstream pathological processes [38, 51].

Since secretase inhibitors have failed repeatedly, the development of therapeutic strategies has shifted to downstream methods aimed at the clearance of pathogenic Aβ species. The most advanced approach in this area is passive immunotherapy by monoclonal antibodies. These antibodies promote amyloid clearance through Fc receptor-mediated microglial phagocytosis, complement system activation, and vascular drainage [4, 76].

#### Epitope specificity and clinical profiles

The efficacy of immunotherapy is highly dependent on the conformation of Aβ targeted by the antibody (Table 1).

**Table 1:** Epitope specificity, mechanisms of action, and clinical outcomes of major anti-amyloid-β monoclonal antibodies evaluated in Alzheimer’s disease

Antibody	Target epitope	Mechanism of action	Clinical outcome (Phase 3)	ARIA-E rate	Route	Status	Ref
Lecanemab (Leqembi)	Soluble protofibrils (residues 1–16, conformational)	Selectively binds toxic protofibrils (> 10 × affinity vs fibrils)	27% slowing (CDR-SB) at 18 months	12.60%	IV and SC	Approved (US, JP, CN, UK). SC approved 2025	[4]
Donanemab (Kisunla)	N3pG Aβ (pyroglutamate, plaques only)	Targets modified Aβ in mature plaques; limited duration dosing	35–36% slowing (iADRS/CDR-SB) in low/med tau (TRAILBLAZER-ALZ 2)	24.00%	IV	Approved (US 2024)	[74]
Remternetug	N3pG Aβ (pyroglutamate)	Highly potent N3pG binder; optimized for SC delivery	Phase 3 ongoing (TRAILRUNNER). Ph1 showed 75% clearance in 6 mos	~ 24% (Ph1)	SC and IV	Phase 3. Fast track	[101]
Trontinemab	Fibrils (gantenerumab + TfR1 shuttle)	Active BBB transport via transferrin receptor (brainshuttle)	Ph1b/2a: > 90% clearance in 6 months. Massive potency increase vs gantenerumab	< 5%	IV	Phase 3 initiating (TRONTIER)	[56]
Aducanumab (Aduhelm)	Aggregated Aβ (residues 3–7)	Binds fibrils/oligomers	Mixed. EMERGE: 22% slowing. ENGAGE: failed	35.00%	IV	Discontinued (Jan 2024)	[32]

### Passive immunization against amyloid-β: mechanistic foundations

Passive immunotherapy comprises the systemic administration of monoclonal antibodies engineered to selectively bind distinct conformational species of Aβ, including soluble oligomers, protofibrils, and insoluble fibrils within senile plaques [6]. In contrast to active immunization, which elicits an endogenous immune response, passive approaches afford precise control over antibody specificity, dosing regimens, and exposure duration; this yields more predictable pharmacokinetic and pharmacodynamic profiles that support tailored therapeutic optimization [104].

**Fig. 2:**
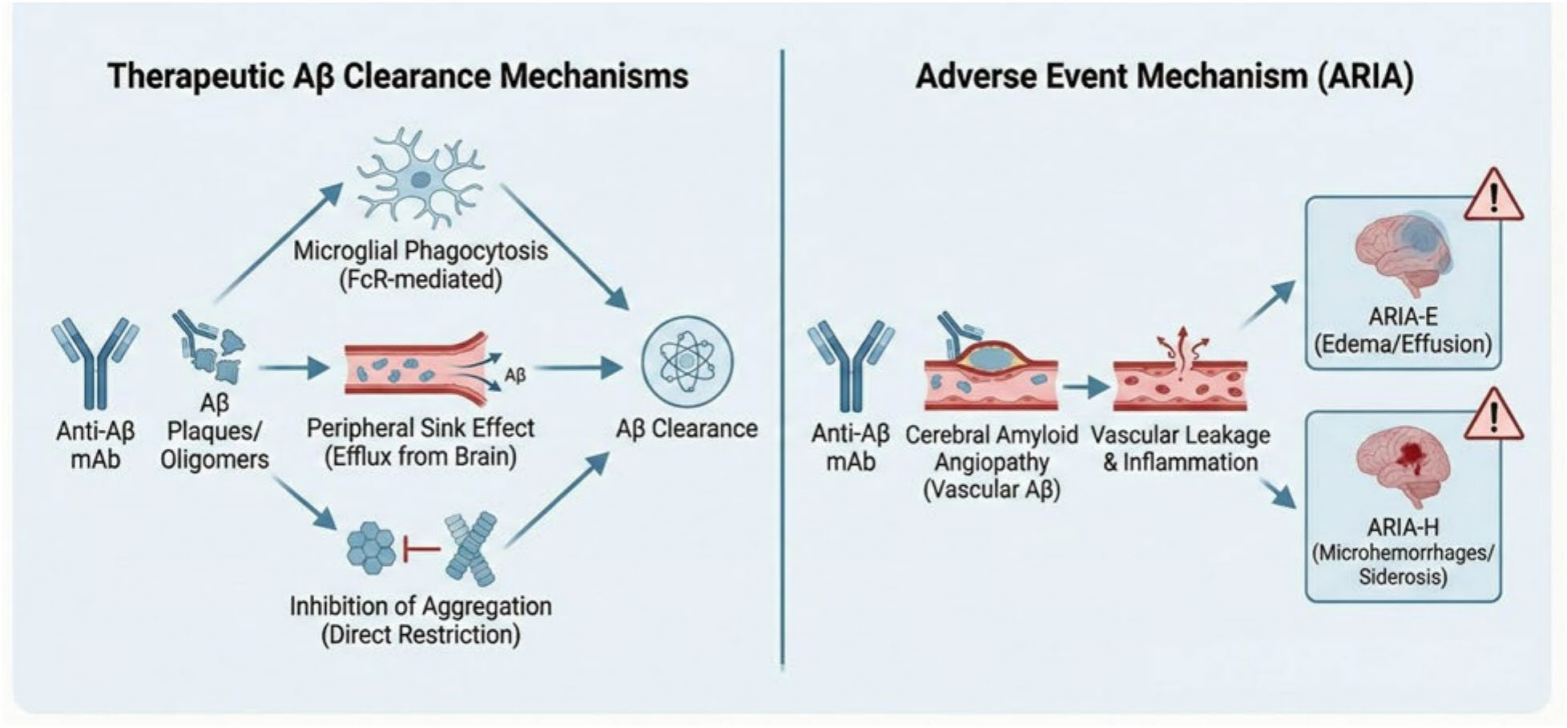
Mechanisms of action and side effects (ARIA)

Several overlapping mechanisms have been recommended to describe the therapeutic actions of anti-Aβ monoclonal antibodies. Antibody binding to cerebral amyloid accumulations can advance microglial activation and Fc receptor-dependent phagocytosis, supporting plaque clearance [144]. In parallel, peripheral sequestration of Aβ may move the equilibrium between central and peripheral compartments, enhancing efflux of soluble peptide from the brain, a phenomenon generally denoted as the peripheral sink effect. Additionally, antibodies targeting aggregation-prone intermediates may directly restrict nucleation and elongation processes, thus reducing the formation and maturation of toxic amyloid assemblies. Especially, differences in epitope recognition, affinity, and isotype among antibodies are increasingly identified as critical determinants of both therapeutic efficacy and adverse event liability [134] **(**Fig. 2**).**

### Clinically advanced anti-amyloid monoclonal antibodies

#### Aducanumab: plaque reduction versus clinical controversy

Aducanumab is a human IgG1 monoclonal antibody with high affinity for Aβ species and, specifically, fibrillar forms present in amyloid plaques. Early clinical trials in humans yielded exciting evidence for antibody-induced plaque reduction, including dose-dependent reductions of cerebral amyloid load as assessed by amyloid positron emission tomography (PET) [26]. Such results positioned aducanumab as an attractive candidate for creating amyloid-blocking medications. However, pivotal phase III trials showed substantial variation in function and cognition. A parallel experiment did not meet its primary effectiveness goal, but one study found a bit of slowing in clinical decline. In doing so, aducanumab has become a stand-in for the larger issues faced by amyloid-aimed treatments, and it has underscored the intricate connections between biochemical changes and treatment results [14].

#### Lecanemab: targeting soluble protofibrils

The preceding argument emphasizes that rationally crafted lecanemab binds to soluble Aβ protofibrils that are increasingly identified as intermediate neurotoxins apt for disrupting synaptic signaling as well as neuronal communication. By specifically targeting these entities (and not fully developed plaques), lecanemab is intended to intervene early in the amyloid aggregation course [75]. According to results from clinical trials, lecanemab comprehensively minimizes brain amyloid levels and significantly slows cognitive decline, though with a discrepancy in statistical relevance versus clinical magnitude. Notably, the uniformity and magnitude of these positive effects have augmented the viewpoint that focused actions on soluble amyloid aggregations may provide an improved balance between efficacy and safety. The above-described scheme has maintained the concept that not all amyloid varieties are equal in disease progression contribution, thus underpinning the need for therapeutic selectivity in conformation [10].

#### Donanemab: rapid amyloid clearance through plaque selectivity

Donanemab identifies a pyroglutamate-adjusted Aβ section present in recognized amyloid plaques. A selective adherence trend enables a treatment period during which many plaques are rapidly and effectively removed. Aggressive plaque removal is correlated to increased treatment-related abnormalities, emphasizing the need for surveillance and patient selection in potent plaque-clearing strategies. Although there are positive cognitive findings for Alzheimer’s treatment, the drug is linked with a higher adverse reaction rate. Healthcare providers must always consider risk and safety when implementing potent approaches. Despite the concerns, removing specific plaques may improve Alzheimer’s disease patients [40].

Although effective in enhancing cognitive abilities, aggressive plaque clearance has been linked to an increased vulnerability to treatment-related imaging abnormalities. Such findings indicate that therapeutic potency and safety are basically at a crossroads, which highlights the significance of selecting patients and undertaking continuous supervision while administering intense amyloid-clearance approaches [29].

### Clinical efficacy end points: interpreting cognitive and functional effects

Valid composite outcome evaluations have been used in clinical research on anti-amyloid monoclonal antibodies to yield data on both cognitive and functional abilities. Some of the most widely used evaluation tools include the Clinical Dementia Rating–Sum of Boxes, which assesses memory, judgment, which is essential for decision-making, and everyday life activities, and the Alzheimer’s Disease Assessment Scale–Cognitive Subscale, which emphasizes memory, language, and praxis. These tools have been demonstrated to be effective in assessing the efficacy of anti-amyloid monoclonal antibodies across clinical trials [110].

In numerous clinical studies, amyloid targeting antibodies have shown a statistically significant slowing of decline on these measures in optimized early AD populations. Conversely, the absolute effect sizes have frequently been small and are often near the margin of clinical significance [31, 123, 124].

### Safety profile and risk management: amyloid-related imaging abnormalities (ARIA)

#### Pathophysiology and classification of ARIA

Amyloid-related imaging abnormalities correspond to the most major and characteristic adverse events associated with passive anti-amyloid immunotherapy. ARIA is classified into ARIA with edema or effusion and ARIA relating to cerebral microhemorrhages or superficial siderosis [60]. These abnormalities are believed to arise from antibody-mediated interactions with amyloid deposits within cerebral blood vessels, leading to transient compromise of vascular integrity and blood–brain barrier function. The incidence of ARIA has highlighted the intimate link between vascular amyloid pathology and therapeutic response, highlighting cerebral amyloid angiopathy as a key modulator of treatment-related risk [55].

#### Risk factors and clinical management

Antibody dose, rate of dose increase, cumulative exposure, and genetic background—specifically carrying the apolipoprotein E ε4 allele—all have an impact on the incidence and severity of ARIA. ARIA events can cause headaches, confusion, dizziness, visual disturbances, or focal neurological deficits. However, many ARIA events are asymptomatic and can only be identified by scheduled magnetic resonance imaging [106].

Current clinical protocols place a strong emphasis on gradual dose titration, routine neuroimaging surveillance, temporary treatment interruption when needed, and customized benefit-risk assessment to reduce these risks. These strategies are now integrated into parts of anti-amyloid treatment initiatives [28].

## Exploring non-amyloid drug targets (the new frontiers)

For many years, scientists have concentrated on a pathway that is triggered by Aβ aggregation, amyloid deposition, and accumulation in the brain as the primary cause of the illness and the most crucial target for treatment [81]. Targeting amyloid in mild-to-moderate patients, as previous unsuccessful clinical studies have done, may not be enough to stop further disease development, as there is growing evidence that amyloid is deposited early in the course of the disease, even before the emergence of clinical symptoms. Other cellular and molecular routes and processes that contribute to AD pathogenesis are being studied by scientists. A combination strategy of therapies that targets two or more parts of pathology (such as amyloid and tau, autophagy and inflammation) may be necessary for successful management, given the disease’s progressively recognized multifactorial nature. As a result, researchers are looking more urgently into other molecular and cellular pathways and processes that contribute to the pathophysiology of AD [67].

### Tau-centric therapies: inhibition of hyperphosphorylation and aggregation

Nominating tau protein as a therapeutic target for AD treatment options has arisen from the dysfunction of this microtubule-associated protein due to the hyperphosphorylation process with consequent intracellular aggregation and formation of neurofibrillary tangles (NFTs) [138]. Furthermore, this hyperphosphorylation-driven tau dysfunction can trigger microtubule instability, disrupted axonal transport, and apoptosis of neurons [70]. This is why NFTs can be a good indicator of worsening and severity of AD [19].

### Neuroinflammation and glial modulation: targeting microglia and astrocytes

Neuroinflammation is a chronic response encompassing activation of glial cells alongside the release of pro-inflammatory elements, leading finally to an inflammatory state in the central nervous system (CNS) [145]. A developing proposition states that neuroinflammation is a fundamental hallmark of the AD pathophysiological scenario [25]. Despite being an initial protective mechanism, prolonged neuroinflammation can exacerbate neuronal damage after initiating over-production of pro-inflammatory cytokines and reactive oxygen species (ROS) as well as complement system activation [65]. The brain immune cells, microglia and astrocytes, exert a protective role in the healthy brain through removal of debris and releasing trophic factors to boost neuronal homeostasis [39, 108]. In terms of AD, these two types of cells display complicated and stage-dependent involvement where activated microglia start to aggregate in the area surrounding the Aβ amyloid plaques constituting a proinflammatory phenotype that is known for its ability to secrete many cytokines such as IL-1β, IL-6 and TNF-α, upon AD progression [49, 88, 130]. It was observed that microglia, during clearance of Aβ amyloid fibrils, show defective phagocytosis [117] and release both ROS and proteases [83, 128, 146], which in turn account for the neurotoxicity of neurons and their synapses [52]. At this stage, a shunt in the cellular state to a disease-associated microglia (DAM) phenotype takes place with distinct distorted transcriptional profiles, greater rate of phagocytosis, and intensified release of proinflammatory cytokines [79, 86]. These activated microglia can also promote downregulation of homeostatic genes and upregulation of phagocytic and lipid metabolism pathways in a manner including two distinct phases: an initial triggering receptor on myeloid cells 2 (TREM2)-independent phase followed by a TREM2-dependent phase [79]. Notably, activated microglia form clusters around amyloid plaques with ingested Aβ fibrils, giving rise to the notion that they curb plaque growth and neurodegeneration [79]. As a consequence, microglial heterogeneity was postulated, where there is adoption of microglia into the DAM state for reducing damage as well as encapsulation of plaques at early stages of the disease, whereas another proposal states that they drive inflammation [7]. This refers to the possibility of a dual role for microglia through either restraining pathology or exacerbating it. Notably, studies using PET revealed that areas with excessive microglial activation are the same areas of tau tangles spreading [143].

Significantly, astrocytes induction is regarded as a crucial factor in the process of neuroinflammation because of its proven involvement in AD neuroinflammatory pathology [7]. Remarkably, reactive astrocytes were found to stop their reinforcing function for homeostasis, and, at the same time, adversely cause deterioration in AD-related inflammation [39, 48, 52]. Mutually, it was also noticed that activated microglia secrete factors (IL-1α, TNF-α, C1q) and therefore, induce the reactive astrocyte subtype A lacking normal helpful tasks and becoming neurotoxic. It is worth mentioning that neurotoxic A1 astrocytes trigger neuronal injury via releasing inflammatory mediators and were perceived in postmortem brains of AD and other neurodegenerative diseases, indicating that this pathological astrocyte conversion occurs in human disease [91]. Consequently, it is reasonable to conclude that a detrimental linkage exists, whereby microglia-guided inflammation drives astrocytes into a neurotoxic A1 state, thereby precipitating neurodegeneration [7]. Importantly, the state of chronic inflammation can promote Aβ amyloidogenesis, since microglia-derived cytokines IL-1α, TNF-α, and C1q can encourage astrocyte-dependent overexpression of β-secretase enzyme with consequent upsurge in Aβ monomer peptide formation [87]. The sustained inflammatory state favors positive feedback: Aβ aggregates stimulate microglia which in turn release neurotoxic elements and enhance development of Aβ aggregates with acceleration to a level that is debilitating and leads to massive worsening of neurological dysfunction [148].

In this milieu, higher AD susceptibility was evident in the case of loss-of-function mutations in TREM2 as they avert its protective role of Aβ phagocytosis, which is mainly related to the TREM2-DAP12 signaling pathway-induced microglial transition to a disease-associated microglial (DAM) phenotype [138]. Thus, there is a new therapeutic strategy with the objective of producing agonistic anti-TREM2 antibodies such as AL002 which are undergoing clinical trials for amplifying microglial resilience [135].

Other glial-targeted therapeutic options encompass the inhibition of CSF1R to restrict microglial proliferation, the inhibition of p38 MAPK and NF-κB to hinder astrocytic inflammation, and cell-based approaches to restore glial function [138]. In addition, transplanting glial progenitor cells in animal models seems to be a promising approach in lessening the burden of Aβ and ameliorating cognition [113].

**Fig. 3:**
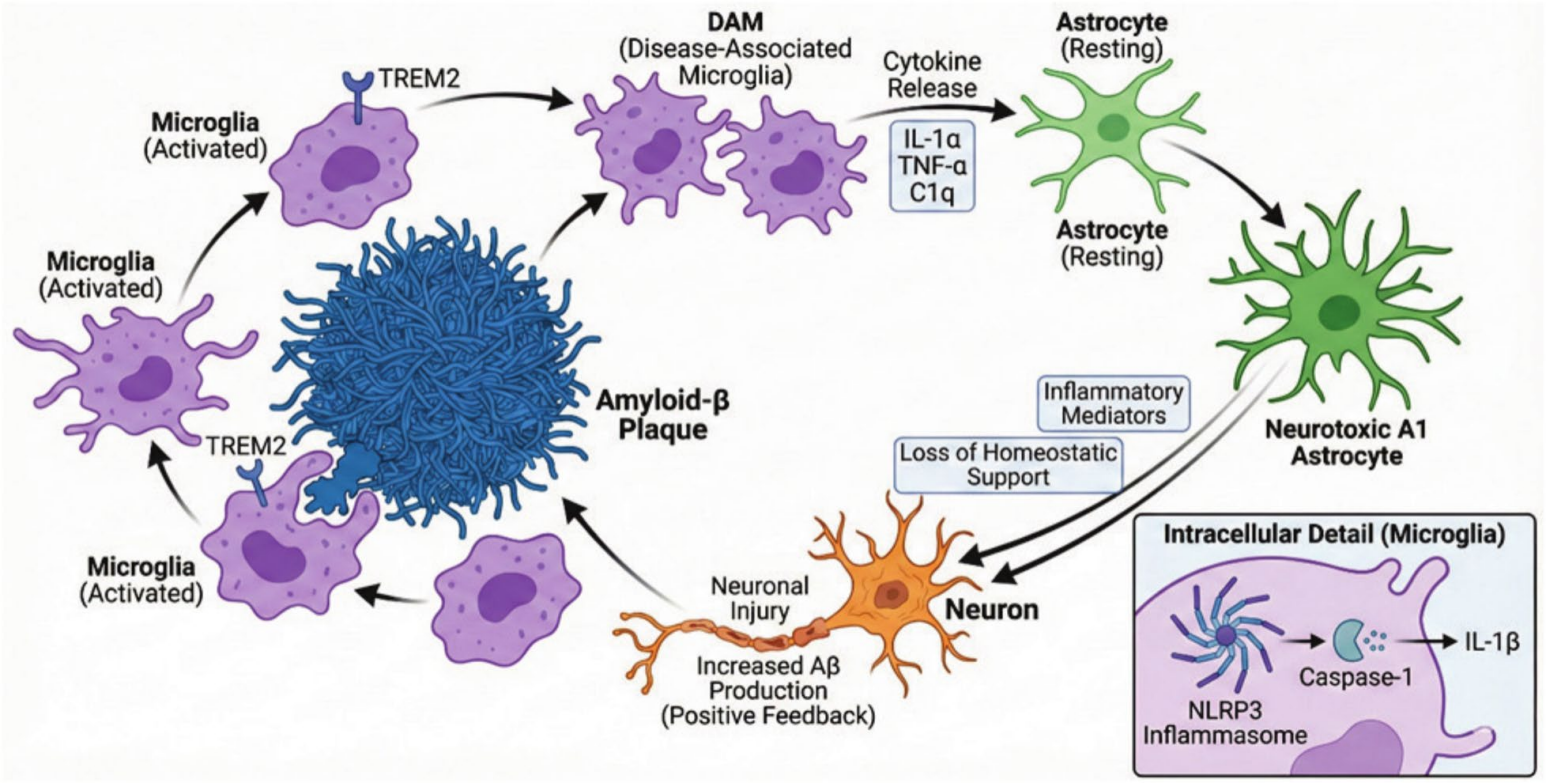
The vicious cycle of neuroinflammation

It was also reported that microglial function is highly dependent on innate immune receptors, such as toll-like receptor 4 (TLR4) and NLR family pyrin domain containing 3 (NLRP3) [138]. This can be verified by induction of nuclear factor kappa light-chain enhancer of activated B cells (NF-κB) signaling and cytokine production in response to TLR4 activation and enhanced caspase-1-mediated IL-1β release with consequent neuronal damage due to NLRP3 inflammasome activation [66]. Accordingly, intervention involving preventing NLRP3 decreases Aβ deposition and cognitive disabilities in AD transgenic models [138] **(**Fig. 3**).**

### Synaptic plasticity and neuroprotection: the role of growth factors and signaling pathways

Previously, the Alzheimer’s Association Research Roundtable (AARR) tackled non-amyloid targets with an emphasis on microtubule (MT) stabilization, which was mostly accomplished by blocking tau phosphorylation. Special attention was paid to the activities of two kinases: cyclin-dependent kinase 5 (CDK5) and glycogen synthase kinase 3-β (GSK3β). By depolymerizing MTs, which disrupts axonal transport and jeopardizes the viability and function of afflicted neurons in AD, tau loss of function may have detrimental effects. Limiting the development of phospho-tau and NFTs was another tau-based strategy. Various non-tau-based approaches include environmental enrichment, the utilization of statins, and anti-inflammatory and antioxidant medications [21].

Emerging evidence suggests that non-amyloid/non-tau (NANT) targets contribute not only to cognitive decline but also to broader clinical manifestations in AD, underscoring their therapeutic relevance [131].

Therapeutics that target beta-amyloid have shown promise in recent clinical studies to decrease the cognitive impairment linked to AD. The most extensively studied NANT biomarker in anti-tau and anti-amyloid therapeutic studies is neurofilament light chain (NfL). The first FDA-approved amyloid mAb, aducanumab, was evaluated in participants with baseline amyloid PET positivity and increased plasma NfL levels [129].

Additionally, the evaluation of NfL showed modest, highly fluctuating modifications, suggesting that NfL was unable to provide information on treatment response. NfL did not alter from baseline to follow-up in trials of tilavonemab, an anti-tau antibody, and lecanemab, an anti-amyloid and anti-protofibril antibody. However, the TRAILBLAZER-ALZ clinical trial, which compared donanemab and aducanumab, showed that the two treatment groups’ plasma NfL increased from the beginning to follow-up. Among anti-amyloid and anti-tau treatment studies, the impact upon additional NANT biomarkers, such as GFAP, neurogranin, and volumetric magnetic resonance imaging (MRI), likewise fluctuated. This is probably due to trial differences, including study design, study duration, baseline, and participant characteristics, as well as underlying heterogeneity of AD [149].

Growth factors (GFs) are involved in the production, maintenance, and repair of neurons and their functionality. Additionally, they facilitate special receptor activation that, in spite of their brief half-life, starts a series of events that activate transcription factors and have long-lasting effects. GFs help maintain brain connections, which may be compromised in hereditary neurodegenerative diseases (hNDDs), by enhancing synaptic plasticity. After a CNS injury, GFs can trigger certain endogenous repair processes. Given all of these traits, along with their ability to reduce inflammation and provide cells with trophic support, GFs are crucial in the fight against hNDDs [105].

Neurogenesis, synaptic plasticity, neuronal development and differentiation, and synaptogenesis are among the functions of a specific protein called brain-derived neurotrophic factor (BDNF) in the CNS. CNS regions like the thalamus, hippocampus, and limbic system are the main sites of BDNF synthesis. Furthermore, immunological cells, skeletal muscles, vascular endothelium, and platelets all produce BDNF peripherally. But the blood–brain barrier (BBB) prevents peripheral BDNF from passing through. The main regions of central BDNF expression are the striatum, frontal cortex, hippocampus, hypothalamus, and midbrain [3].

BDNF is a growth factor, involved in the basal forebrain bundle’s cholinergic nerve cells’ consistency and effectiveness. AD is known to cause diminished memory and cognitive impairment due to the distortion of cholinergic neurons in the basal forebrain bundle. The underlying cause of AD is connected to impairment of BDNF signaling, according to several findings. The most abundant neurotrophin in the central nervous system, BDNF, is essential for both neuronal survival and synaptic plasticity. By blocking the amyloidogenic pathway and lowering the production of neurotoxic Aβ in transgenic mice, BDNF has a neuroprotective function against the pathogenesis of AD by decreasing the expression and activity of β-secretase. Furthermore, in transgenic mice, BDNF increases α-secretase expression and activity, which leads to the synthesis of the neuroprotective soluble APP alpha (sAPPα) [114].

BDNF has emerged as a major target in the physiopathology of several mental and neurological disorders. Potential cognitive deterioration in healthy elderly individuals is predicted by altered levels of BDNF in cerebrospinal fluid (CSF) and the blood circulation in people with AD. It has very negative effects when compared to other neurotrophic factors like ciliary neurotrophic factor (CNTF), which enhances synaptic plasticity and cognitive impairment in mice. When it comes to reducing cognitive impairment, BDNF works better than CNTF. Additionally, people with AD or cognitive impairment had lower blood concentrations of BDNF [107].

BDNF plays a crucial role in mediating both activity-dependent and activity-mediated plasticity as well as synaptic remodeling that takes place in response to developmentally contextualized environmental cues. Experience-dependent plasticity is a crucial developmental mechanism that fine-tunes brain circuitry and the sensorimotor abilities that follow. By promoting the migration of synaptic proteins (AMPA receptors, synapsin, and PSD-95) to active locations and enabling TrkB signaling to stabilize developing synaptic connections, BDNF promotes synaptic development. Actin polymerization may also alter dendritic spine density and architecture, improving synaptic performance [72].

Reintroducing BDNF has been shown in animal experiments to restore plasticity in visual circuits, enabling amblyopia recovery. Additionally, it has been demonstrated that early sensory input can strengthen and improve synaptic discrimination as well as advance BDNF expression in somatosensory circuits during development. It has also been demonstrated that BDNF overexpression throughout adolescence enhances prefrontal cortical plasticity in non-human animals. Higher-order cognitive tasks may be supported by the prefrontal cortex, a crucial brain route for controlling emotions and making decisions [122].

#### Synaptic plasticity

Synaptic plasticity is the regulation of synaptic power, principally via LTP and long-term depression (LTD). LTP, which is well recognized as a critical chemical basis for learning and memory, improves synaptic efficacy by increasing receptor density and neurotransmitter release at active synapses. Continuous practice or repetition of a skill solidifies these enhanced connections, reinforcing the brain circuits concerned. For example, an adaptive form of plasticity arises when synaptic connections are reinforced during skill development, such as practicing a musical instrument, or during long-term memory formation, such as acquiring a new language [109].

#### BDNF and synaptic plasticity: mechanisms of long-term potentiation (LTP) and long-term depression (LTD)

**Fig. 4:**
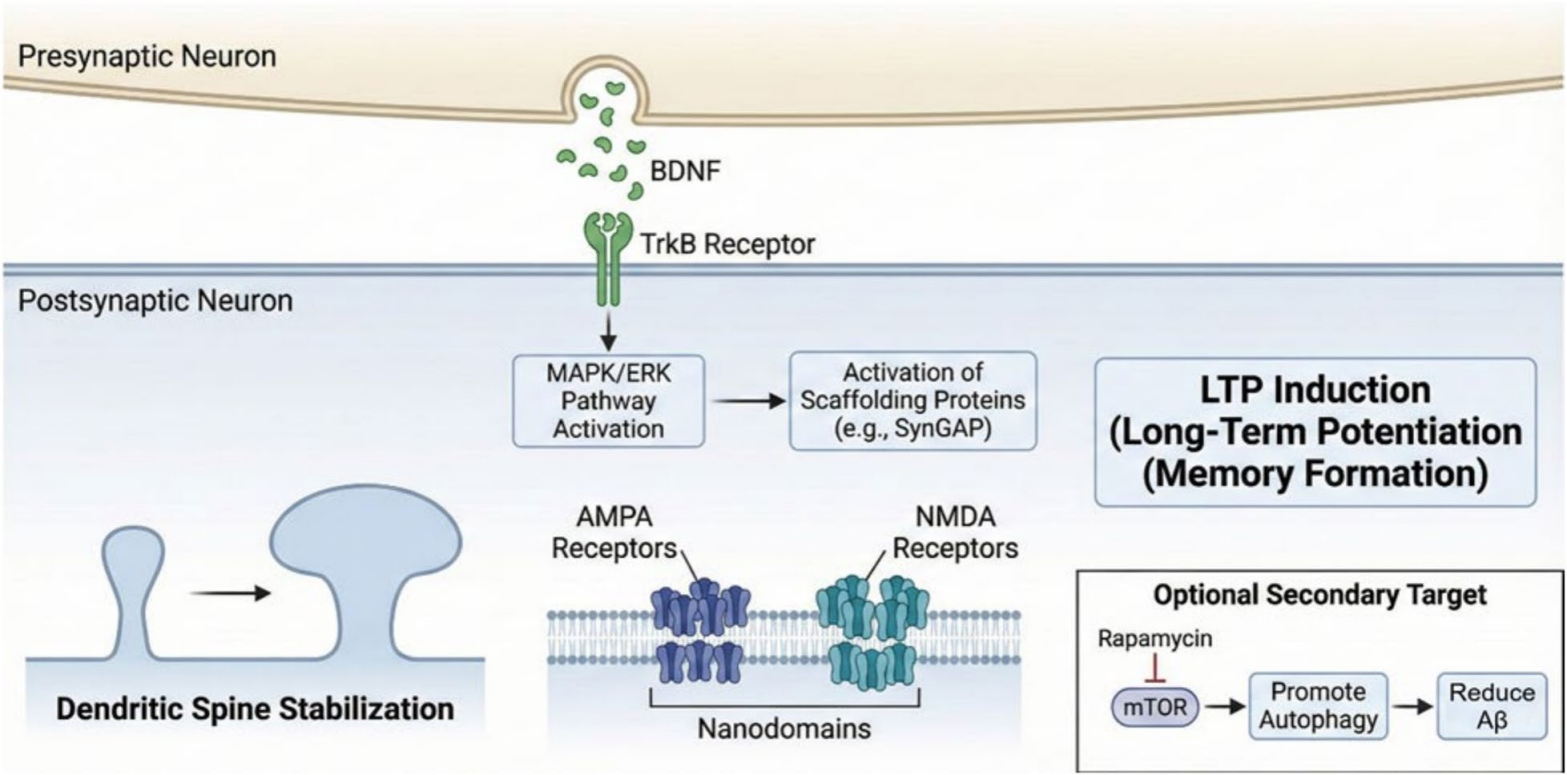
Non-amyloid targets: BDNF and synaptic plasticity

BDNF, a neurotrophic factor linked to learning and memory as well as behavior adaptation, is a key element of the mechanisms involved in LTP and LTD, both of which are synaptically specific in processing learning and memory while also allowing neural circuits to balance and adjust to environmental expectations. LTP mechanisms include the following: LTP is an extended rise in synaptic efficacy (outlasting the stimulus) caused by a pattern of prolonged stimulation that is thought to be dependent on BDNF. BDNF binds to TrkB receptors, which activate the MAPK/ERK signaling cascade, resulting in the activation of scaffolding proteins (e.g., SynGAP), which upregulate receptor clustering and stabilize dendritic spines [147] **(**Fig. 4**).**

Mechanisms of LTD: LTD decreases synaptic performance, allowing the brain network to function more efficiently by eliminating unnecessary or superfluous connections. For instance, BDNF uses clathrin-dependent pathways to modulate AMPA receptor endocytosis, which in turn induces LTD. The neurotrophin receptor p75’s interaction with ProBDNF, which triggers JNK-dependent signaling pathways that alter the cytoskeleton and retract the dendritic spines, mediates the mechanism’s intricacy. There is proof that these processes involve BDNF-dependent LTD. This type of plasticity can aid in the early formation of hippocampal circuits as well as prevent overconnectivity by permitting synaptic homeostasis [118].

BDNF also promotes the local translation of numerous key plasticity-related genes, including Arc, by attracting ribonucleotide granules to active dendritic spines. BDNF additionally arranges spines to AMPA and NMDA receptors, forming nanodomains that serve as efficient signaling regions. When seen with sophisticated two-photon microscopy, BDNF aggregates AMPA and NMDA receptors as nanodomains at spines to produce effective signaling zones. According to optogenetic investigations, whether BDNF is released during theta-burst activation impacts the volume of calcium flux that enters the cell, which directly correlates synaptic potentiation with the availability of BDNF [23].

A prominent member of the insulin-like growth factor family, insulin-like growth factor 1 (IGF-1) is a 7.6 kDa peptide made up of 70 amino acids. To investigate the neuroprotective potential of IGF-1, observations of disturbed IGF-1 signaling in AD patients—characterized by a decreased active/inactive IGF-1 ratio and increased IGF-1R expression—were made. Consequently, IGF-1 transduction was discovered to prevent memory impairment in AD mice, indicating that increasing IGF-1 expression in the brain may be a potential defense against AD-related neuronal death and memory loss [127].

VEGF-A is primarily recognized for its involvement in vasculogenesis, angiogenesis, and neuroprotection, among other roles. Endothelial cells involved in angiogenesis and barrier permeability, as well as glial cells and neurons, are primary sources in the central nervous system. VEGF-A, a GF, has been shown to increase synaptic plasticity, impacting memory and learning processes. VEGF-A has also been shown to boost neuronal survival by promoting angiogenesis and lowering neuroinflammation. As a result, increasing VEGF-A levels may protect neurons from injury and improve survival pathways. VEGF-A has been shown to have a regenerative effect on both the central and peripheral nervous systems (PNS). VEGF-A has been linked to increased axonal development and Schwann cell activation, both of which are critical for brain connection rehabilitation and restoration [44].

### Vascular and metabolic targets

Non-amyloid/non-tau (NANT) targets, besides being neurotransmitters and neuroprotective, also affect vascular and metabolic pathways, inflammation, and synaptic plasticity [17]. The glucagon-like peptide-1 (GLP-1) receptor agonist semaglutide, which has been utilized extensively for the prevention of type 2 diabetes, has recently attracted attention as a possible NANT-focused treatment for AD. The brain has a high expression of the GLP-1 receptor, and it has been demonstrated that GLP-1 contributes significantly to the pathogenesis of AD through several neuroinflammatory, vascular, and other pathological mechanisms. Therefore, GLP-1 receptor agonists may be interesting treatment candidates to delay the course of AD by modulating neuroinflammation and possibly reducing neurodegeneration through a variety of modes of action. Furthermore, patients with type 2 diabetes who take semaglutide are half as likely to get dementia over the course of their lifetime, which highlights the drug’s potential clinical usefulness in AD [2].

Masitinib is a small-molecule medication taken orally that specifically inhibits the tyrosine kinase c-kit. This modulation of mast cell survival, differentiation, and degranulation results in the control of inflammatory mediators. Masitinib restored synaptic markers and spatial learning abilities in AD [90].

Canakinumab is a humanized monoclonal antibody that inhibits IL-1β. It is a proinflammatory cytokine that controls a variety of innate immunity-related inflammatory reactions, making it a master regulator of inflammation. IL-1β buildup was seen surrounding Aβ plaques in postmortem AD patient brains, and it is linked to neurodegeneration and inflammation-related neuronal injury [71].

Rapamycin inhibits the mechanistic target of rapamycin (mTOR), a protein that regulates metabolism, proliferation, differentiation, migration, and dendritic formation, among other cellular functions. Glycogen synthase kinase 3β (GSK3-β) contributes to the AD pathogenesis of tau and Aβ and is engaged in the mTOR pathway. Rapamycin is suggested as a treatment for AD that targets GSK3-β via the mTOR pathway. Rapamycin has been shown in preclinical experiments to mitigate Aβ plaque load, decrease β- and γ-secretase expression, boost Aβ clearance through autophagy, and reduce pTau by upregulating an enzyme that breaks down insulin [24].

## Clinical trial methodology and outcomes assessment

The era of disease-modifying therapies has necessitated a fundamental shift in clinical trial methodology. Historically, trials recruited patients based on clinical symptoms, often resulting in heterogeneous cohorts. Current protocols, driven by the 'biological definition' of AD, now mandate biomarker confirmation for enrollment, moving the focus from symptomatic relief to target engagement [137]. Biofluids including CSF and plasma, as well as imaging modalities like brain MRI or 18F-fluorodeoxyglucose (FDG)-PET scans of the brain, can be used to assess these biomarkers [96]**.** Following this, research has demonstrated that new medications can reduce brain amyloid levels, which are linked to a slight improvement in cognitive function when compared to a placebo group. However, further investigation is necessary due to the side effects linked to these medications [136]**.**

Thus, it is crucial to comprehend the processes of AD early on, when the disease is most amenable to treatments that can change its trajectory. As significant as AD biomarkers are for evaluating current pathology, it may also be useful for risk assessment to test for genes linked to familial Alzheimer's disease (FAD) or late-onset Alzheimer's disease (LOAD). Presenilin 1 and 2 (PSEN1 and PSEN2) are implicated in 80% of FAD cases due to extremely rare and penetrant autosomal dominant mutations. Typically, symptoms do not appear until well before the age of sixty. Further 10%–15% of cases are due to APP mutations on chromosome 21q21, incredibly rare are mutations in PSEN2 on chromosome 2q31-q42, and at least 50% of cases are due to mutations in PSEN1 on chromosome 14q24.3 [73]**.** Most cases of AD occur in people who develop the condition later in life, and this is due to the polygenic risk that is caused by several susceptibility genes. Although more than 40 risk alleles for AD have been found by genome-wide association studies, the APOE gene on chromosome 19q13.2 carries a substantially higher risk than other locations and accounts for 20%–29% of AD cases [78]. Thanks to its three distinct allelic variations, APOE can generate the following potential genotypes: ε2/ε2, ε2/ε3, ε2/ε4, ε3/ε3, ε3/ε4, and ε4/ε4. People with the ε3/ε4 genotype have a 2 to threefold increased chance of getting AD, whereas those with the ε4/ε4 genotype can have a risk that is up to 15 times greater [53]**.**

A population-based technique that is both cost-effective and non-invasive is one option, while a focused approach that identifies the at-risk group is the other. Both methods can be used to determine who is at risk for dementia. The second option may be more costly, invasive, and inconvenient since it concerns a smaller group of people. In order to identify individuals who require more expensive testing and to provide them with tailored treatment or preventative measures, population-based screening utilizing biomarkers found in blood could be beneficial [125]**.** Current US Preventive Task Force (USPSTF) recommendations do not endorse population-based screening for cognitive impairment in older persons because more study is needed to weigh the advantages and risks of this practice [116]**.** The following is in line with Sackett’s guideline: "screening for a disease is appropriate when available screening tests are acceptable to patients, when early treatment is more advantageous than later intervention, and when time and resources permit screening, diagnosis, and treatment of the condition." [8]**.**

### The crucial role of biomarkers in patient selection and monitoring

When it comes to AD clinical trials, biomarkers have been gamechangers. In the past, clinical complaints were the only criteria for patient enrollment, which resulted in a diverse study group and higher failure rates. Now, biomarkers play an essential role in the pathological diagnosis of AD, the identification of suitable patients, the monitoring of disease progression, and the evaluation of therapeutic efficacy, especially for treatments that modify the illness.

To aid in the identification, differentiation, and diagnosis of AD phenotypes, the most common biomarkers for the disease can be grouped into two types: pathophysiologic and topographic. Amyloid PET, levels of amyloid and tau proteins in CSF, and levels of these and other protein biomarkers in plasma are all pathophysiological biomarkers linked to lesions in AD. Imaging techniques such as brain FDG-PET and tau PET reveal regional hypometabolism, which is linked to topographic biomarkers and the localized effects of AD pathology [42]**.**

Diagnostic biomarkers are measurable indicators of illness presence, progression, and underlying pathogenic processes. To improve diagnosis accuracy, monitor disease progression, and assess treatment effectiveness, biomarkers play a vital role in AD. Some AD biomarkers are found in CSF, whereas others are found in blood [76]**.**

### Cerebrospinal fluid markers

Encasing the central nervous system and brain is a clear liquid called CSF. Cognitive impairments associated with metabolic changes in CSF can be better understood through biomarker analysis of CSF [9]**.** Markers indicating AD in cerebrospinal fluid that are most widely acknowledged are:

The main components of amyloid plaques, known as Aβ peptides, include two notable variations at the C-terminus: AŲ40 and AŲ42. The main variety is Aβ40, whereas Aβ42 is more likely to form oligomers and fibrils in the extracellular environment [76]**.** The amount of Aβ plaques in the brain is negatively correlated with Aβ42, the main biomarker. The number of Aβ plaques increases as the concentration of Aβ42 drops. The Aβ42/Aβ40 ratio shows great diagnostic effectiveness and is used to assess the probability of AD development [58]**.**

#### Tau proteins

In 1993, the first possible biomarker was found to be an increased total tau level in cerebral fluid [84]**.** Microtubule stabilization and neuronal damage severity are both indicated by the tau protein. Overly hyperphosphorylated (p-tau) in AD is an essential component of neurofibrillary tangles. Several p-tau biomarkers have been discovered, such as p-tau181, p-tau217, and p-tau231. These indicators are useful for AD staging and differential diagnosis [142]**.**

Each component of the Aβ42/Aβ40 ratio, total tau, and p-tau provides unique pathophysiological features that can be used to predict the pathology of AD in cerebrospinal fluid. Specifically, Aβ42/Aβ40 represents amyloidosis, total tau represents neurodegeneration, and p-tau represents neurofibrillary tangles. It was determined that these signs appeared several years before the disease really manifested. Changes in Aβ and p-tau concentrations in the CSF were documented by McDade et al. in patients with dominantly inherited AD, 25 and 10 years before dementia onset [99]**.**

Additional CSF markers that are currently under development include astrocyte activation (YKL-40) and microglial activation (TREM-2, TSPO) [20]**.** The study of cerebrospinal fluid is very intrusive and puts a burden on patients, although biomarkers in this fluid provide insights into the pathogenesis of disease. Therefore, finding biomarkers for early AD that do not involve invasive procedures or those that are just mildly invasive is a growing area of study focus [63]**.** In order to create biomarkers, researchers are looking at a variety of physiological fluids, such as saliva, urine, blood, and tears.

The non-invasive nature and easy accessibility of blood-based biomarkers make them highly attractive for use in the diagnosis and monitoring of AD. Some intriguing options have arisen [137]**,** including the following, although research and development of blood-based biomarkers continue:

Potential markers of AD have been explored in relation to plasma Aβ and tau protein levels, and the ratio of plasma Aβ42/40 is comparable to biomarkers discovered in the cerebrospinal fluid. Unlike the CSF ratio, which drops by 50%, the plasma Aβ42/40 ratio drops by only 10–15%. Their efficacy is still up for discussion, and more study is required to establish the diagnostic accuracy of these approaches [57]**.**

Phosphorylated tau levels in cerebrospinal fluid are comparable to those in plasma; both are elevated in AD patients. Threonine 231 is the site of the first phosphorylation in AD, with further increases at threonine 217, 218 and 205 [89]**.**

Increased levels of the protein neurofilament light chain (NfL), which is released into the bloodstream during neuronal injury, have been linked to neurodegeneration and cognitive loss in AD [1]**.** Patients with AD had 2.61 times greater blood NfL levels compared to controls; nevertheless, there was no statistically significant difference in plasma NfL levels [62]**.**

When it comes to intermediate filament III proteins, astrocytes have glial fibrillary acidic protein III (GFAP). Plasma GFAP levels increase during the progression of AD, according to multiple investigations. They can be used as early indicators of AD and are common in people with both preclinical and symptomatic forms of the disease [47]**.**

### PET markers

By employing a variety of ligands in PET imaging, medical professionals are better able to distinguish between typical and unusual forms of AD, which helps in the differential diagnosis of other neurological diseases.

To distinguish between typical and abnormal cases of AD, FDG-PET can be utilized as a topographic biomarker by analyzing patterns of regional hypometabolism that are suggestive of clinical abnormalities across different types of the illness [54]**.** One characteristic of AD is bilateral hypometabolism in the parietal and medial temporal areas, which include the precuneus**.** By exposing hypometabolic patterns, this imaging technique helps differentiate AD from other types of dementia, including frontotemporal dementia (FTD) and dementia with Lewy bodies [102]**.** Not as many people use FDG-PET as MRI, even though it provides better diagnostic information.

In research settings, amyloid PET is mostly used to see insoluble or fibrillar Aβ plaques in the brain, not other types of the Aβ peptide [59]**.** In imaging-to-autopsy investigations, amyloid PET has been shown to be the most accurate biomarker for detecting amyloid plaques, with a sensitivity of 92% and a specificity of 100% [27]**.** Patients with atypical and traditional AD have similar amyloid distributions when using PET, in contrast to other imaging modalities [42]**.** Unlike fluid markers, Tau PET, which shows neurofibrillary tangles, is mainly used for research. It is the only direct marker of neurofibrillary tangles that has been verified by autopsy (sensitivity, 92–100%; specificity, 52–92%). Unlike amyloid PET, tau PET has shown promise in predicting cognitive decline in individuals without impairment. It is clinically important since its evaluation corresponds to short-term progression (3–5 years) in this group [115]**.** Topographic tau PET, in contrast to amyloid PET, more faithfully depicts the clinical phenomenology through its deposition patterns [42]**.**

Individuals with logopenic aphasia, medial temporal regions in individuals with the amnestic type of AD, and occipital regions with posterior cortical atrophy all show higher Tau PET ligand binding [42]**.** Differences in Tau PET patterns between atypical AD and the normal (amnestic form) may help shed light on the molecular basis of these two phenotypes.

Other PET biomarkers that are currently under investigation to aid in the diagnosis of AD are translocator protein PET, which evaluates neuroinflammation, and synaptic vesicle glycoprotein 2 A PET, which evaluates synaptic density [100]**.** These markers serve as tools for disease staging and prognosis instead of differential diagnosis. However, these PET tracers are currently underutilized in clinical settings.

### Integration of fluid and imaging biomarkers for early evaluation

The combination of fluid biomarkers (CSF, plasma) and imaging biomarkers (PET, MRI) has revolutionized the conduct of AD clinical trials from symptomatic to more biologically driven research. The combination is critical to early evaluation, which can often be used as a surrogate marker for benefit prior to the onset of functional symptoms [46, 144].

CSF biomarkers remain the gold standard for the earliest identification. The Aβ42/40 ratio and the p-tau181 have the highest sensitivity and specificity to pathology [11, 103, 119]. They form the basis of target engagement.

Plasma biomarkers: The development of blood biomarkers, especially p-tau217 in plasma [11, 103, 119], represents a revolution in accessibility. These markers show high correlation with CSF and PET findings and are increasingly used for screening [92], allowing for non-invasive identification of amyloid pathology.

Imaging (PET): Amyloid PET allows visualization and quantification of plaque extent in centiloids. This is the main proof of mechanism, where the effect size is "very large," and about the same degree of amyloid deposition is reduced by 35–85 centiloids in the cases of donanemab and lecanemab [11, 22, 144].

#### Efficacy-biomarker discrepancy

The "Efficacy–biomarker Discrepancy" represents the primary issue dealt with in existing literature. Efficacy–biomarker discrepancy refers to a large discrepancy existing between the size of target engagement and the size of clinical benefits.

Tau independence: According to this report, tau pathology, after its initiation, may be a consequence of a separate process from amyloid. Thus, an amyloid cleanse in a patient who has established spread of tau pathology may be inadequate [22, 119].

Neurodegeneration as a rate limiter: Structural damage in the form of atrophy is a permanent thermodynamic barrier. For most patients with substantial underlying levels of atrophy, little clinical advantage can be achieved beyond the removal of amyloid [11, 119]. This is because the underlying neuronal substrate supporting cognitive function has already been.

Cognitive reserve: The heterogeneity in cognitive reserves (education and genetics) can mask the treatment effects. Individuals with high reserves can function normally despite the pathology, whereas individuals with poor reserves will deteriorate irrespective of the treatment, thereby diluting the observed effects in mixed populations entering trials [45, 136].

#### Challenges in interpreting clinical efficacy: the need for meaningful change

The need for meaningful change is the primary challenge in interpreting the efficacy of these novel therapies. It is up to the medical community to determine what constitutes meaningful change in patient benefit versus substantial risk and cost.

The measurement of meaningful change: Statistically significant change on a scale is not sufficient. Meaningful change can be described as the preservation of function sufficient for the patient to "maintain their independence, identify their family, or engage in daily activities for more than they would have been able without the treatment." Regarding the present effect sizes on CDR-SB, there appears to be doubt as to whether these medications represent “meaningful change [11, 45].

Benefit–risk ratio: The limited benefits have to be balanced against the clinically important harms identified as ARIA [41]. The systematic reviews have documented the high level of increased risks of brain edema and hemorrhage associated with the treatment [92, 144]. The physician is confronted with the ethical problem of administering a drug that reduces a biomarker and results in limited cognitive slowing versus potential serious side effects [43, 136].

## Future directions and conclusion

### Combination therapies: the necessity of a multi-target approach

The small effect sizes that were seen with anti-amyloid monotherapies imply that although amyloid clearance is a vital first step, it is probably not enough to completely halt disease progression alone. The complex pathophysiology of AD, including the propagation of tau, long-term neuroinflammation, synaptic dysfunction, and vascular dysregulation, requires a paradigm shift to combinatorial therapeutic strategies. Future clinical trials will need to investigate "cocktail" strategies that will combine anti-amyloid agents with downstream effectors. For example, combining an amyloid-clearing antibody with an inhibitor of tau aggregation could theoretically stop both the trigger, or cause, of neurodegeneration (Aβ) and the driver (tau) of neurodegeneration. Similarly, combining anti-inflammatory agents to alter microglial phenotypes such as TREM2 agonists may help maintain synaptic integrity in an amyloid-free brain. This multi-modal approach is modeled on successful paradigms in oncology and HIV because AD is not a single-proteinopathy, but rather a multifaceted failure of proteostasis and immune regulation.

### Precision neuropharmacology: defining patient subtypes and targeted treatment

The era of ‘one-size-fits-all’ AD treatment is ending; the future is in precision neuropharmacology, where therapeutic choice is determined by the specific biological nature of a patient (rather than a generalized clinical diagnosis). Biomarker profiling has now made it possible to stratify patients into biological subtypes. For example, patients with dominant amyloid pathology and little spread of tau may benefit the most from aggressive plaque clearance, while those with advanced neuroinflammation or metabolic dysregulation may benefit more from glial modulators or insulin-sensitizing compounds such as GLP-1 agonists. This shift is a method to ensure that the right drug is delivered to the right patient at the right biological stage so that not only is efficacy maximized but exposure to drugs that are not efficacious is minimized.

### How genetics and individual pathology will guide future therapy selection

Genetics and individual pathology will have a definitive role in therapy selection in the future, especially as far as safety and risk management are concerned. The strongest genetic risk factor for sporadic AD, as well as a critical predictor of ARIA, is the APOE ε4 allele. APOE ε4 homozygotes have a significantly increased risk of developing severe ARIA on immunotherapy; thus, it is possible that in the future, protocols will need to be tailored to suit everyone, with a higher dose for these high-risk carriers and/or even prioritizing them for non-amyloid treatments to prevent vascular complications. Furthermore, pathological staging using tau PET as well as fluid biomarkers will help with better patient selection by defining "therapeutic windows". Evidence seems to indicate that once tau pathology becomes self-propagating, the effects of amyloid removal are decreasingly efficacious, which brings us to the idea that individual pathology will determine the order of therapeutic intervention: early-stage patients will receive anti-amyloid induction, whereas later-stage patients will need immediate neuroprotective or synaptic support.

### Conclusion

The approval of disease-modifying anti-amyloid antibodies represents an important inflection point in the history of neuropharmacology because it supports the long-held hypothesis that the pathology of AD can be altered. However, this success is not a cure, but a portal to a higher understanding of the disease. The "new era" of AD treatment will be characterized not by a single "magic bullet," but by a whole arsenal of targeted therapies. By combining combination strategies, precision medicine, and stringent genetic risk stratification, the field can shift its focus from simply slowing decline to significantly preserving quality of life in millions of patients worldwide.

## Data Availability

Data are available within the article.

## Abbreviations

AD: Alzheimer’s disease
ADAS-Cog: Alzheimer’s Disease Assessment Scale–Cognitive Subscale
APP: Amyloid precursor protein
ARIA: Amyloid-related imaging abnormalities
ARIA-E: ARIA with edema or effusion
ARIA-H: ARIA relating to cerebral microhemorrhages or superficial siderosis
Aβ: Amyloid-beta
Aβ40: Amyloid-beta peptide 40
Aβ42: Amyloid-beta peptide 42
BACE1: β-Site APP-cleaving enzyme 1
BBB: Blood–brain barrier
BDNF: Brain-derived neurotrophic factor
CDK5: Cyclin-dependent kinase 5
CDR-SB: Clinical Dementia Rating–Sum of Boxes
CHL1: Close homolog of L1
CSF: Cerebrospinal fluid
DAM: Disease-associated microglia
FAD: Familial Alzheimer's disease
FDG-PET: 18F-fluorodeoxyglucose positron emission tomography
GFAP: Glial fibrillary acidic protein
GLP-1: Glucagon-like peptide-1
GSK3-β: Glycogen synthase kinase 3-β
GSMs: γ-Secretase modulators
IGF-1: Insulin-like growth factor 1
IL-1β: Interleukin-1β
IL-6: Interleukin-6
LOAD: Late-onset Alzheimer's disease
LTD: Long-term depression
LTP: Long-term potentiation
MRI: Magnetic resonance imaging
NANT: Non-amyloid/non-tau
NF-κB: Nuclear factor kappa-light-chain-enhancer of activated B cells
NLRP3: NLR family pyrin domain containing 3
NRG1: Neuregulin-1
NfL: Neurofilament light chain
PET: Positron emission tomography
PSEN1: Presenilin 1
PSEN2: Presenilin 2
ROS: Reactive oxygen species
SEZ6: Seizure protein 6
TLR4: Toll-like receptor 4
TNF-α: Tumor necrosis factor-α
TREM2: Triggering receptor on myeloid cells 2
TfR1: Transferrin receptor
VEGF-A: Vascular endothelial growth factor A
iADRS: Integrated Alzheimer's Disease Rating Scale
mTOR: Mechanistic target of rapamycin

## References

[1] Abed SS, Hamdan FB, Hussein MM, Al-Mayah QS (2023) Plasma tau and neurofilament light chain as biomarkers of Alzheimer’s disease and their relation to cognitive functions. J Med Life 16:284 36937471 10.25122/jml-2022-0251 PMC10015560

[2] Akimoto H, Negishi A, Oshima S, Wakiyama H, Okita M, Horii N, Inoue N, Ohshima S, Kobayashi D (2020) Antidiabetic drugs for the risk of Alzheimer disease in patients with type 2 DM using FAERS. Am J Alzheimers Dis Other Demen 35:1533317519899546 32162525 10.1177/1533317519899546 PMC11005324

[3] Ali NH, Al-Kuraishy HM, Al-Gareeb AI, Alnaaim SA, Alexiou A, Papadakis M, Saad HM, Batiha GE-S (2024) The probable role of tissue plasminogen activator/neuroserpin axis in Alzheimer’s disease: a new perspective. Acta Neurol Belg 124:377–388 37917293 10.1007/s13760-023-02403-x PMC10965687

[4] Alkhalifa AE, Al Mokhlf A, Ali H, Al-Ghraiybah NF, Syropoulou V (2025) Anti-amyloid monoclonal antibodies for Alzheimer’s disease: evidence, ARIA risk, and precision patient selection. J Pers Med 15:437 41003140 10.3390/jpm15090437 PMC12470750

[5] Almeida ZL, Vaz DC, Brito RM (2025) Morphological and molecular profiling of amyloid-β species in Alzheimer’s pathogenesis. Mol Neurobiol 62:4391–4419 39446217 10.1007/s12035-024-04543-4 PMC11880078

[6] Alshamrani M (2023) Recent trends in active and passive immunotherapies of Alzheimer’s disease. Antibodies 12:41 37366656 10.3390/antib12020041 PMC10295010

[7] Amniouel S, Suh J, Zheng W, Zhang Q (2025) Beyond amyloid: nanobody-mediated neuroinflammatory therapy for Alzheimer’s disease. Transl Neurodegener 14:51 41084027 10.1186/s40035-025-00513-5 PMC12516901

[8] Angelidou IA, Makri M, Beyreuther K, Boada Rovira M, Despoti A, Engelborghs S, Miguel A, Rodríguez I, Stocker H, Temmerman J (2023) Attitudes toward pre-symptomatic screening for Alzheimer’s dementia in five European countries: a comparison of family members of people with Alzheimer’s dementia versus non-family members. Front Genet 14:1305107 38162684 10.3389/fgene.2023.1305107 PMC10757380

[9] Anoop A, Singh PK, Jacob RS, Maji SK (2010) CSF biomarkers for Alzheimer′ s disease diagnosis. Int J Alzheimers Dis 2010:606802 20721349 10.4061/2010/606802 PMC2915796

[10] Ashmawy RE, Okesanya OJ, Ukoaka BM, Daniel FM, Ezedigwe SG, Agboola AO, Ahmed MM, Ogaya JB, Amisu BO, Adigun OA (2025) Exploring the efficacy and safety of lecanemab in the management of early Alzheimer’s disease: A systematic review of clinical evidence. J Alzheimer’s Disease 105(3):13872877251331640 10.1177/13872877251331640 40232258

[11] Avgerinos KI, Manolopoulos A, Ferrucci L, Kapogiannis D (2024) Critical assessment of anti-amyloid-β monoclonal antibodies effects in Alzheimer’s disease: a systematic review and meta-analysis highlighting target engagement and clinical meaningfulness. Sci Rep 14:25741 39468148 10.1038/s41598-024-75204-8 PMC11520896

[12] Bano S, Raza MA, Ghosh S, Pandit NS, Srivastava S, Azam M, Dey SK, Han SS, Sinha JK, Ruwali M (2023) Emerging therapeutic targets in molecular neuropharmacology for Alzheimer’s disease. Biol Regul Homeost Agents 37:5769–5784

[13] Bayraktaroglu I, Ortí-Casañ N, Van Dam D, De Deyn PP, Eisel UL (2025) Systemic inflammation as a central player in the initiation and development of Alzheimer’s disease. Immunity Ageing 22:33 40841660 10.1186/s12979-025-00529-5 PMC12369153

[14] Behl C (2023) Driving Forces of Alzheimer’s Research Directions. In: Alzheimer’s Disease Research: What Has Guided Research So Far and Why It Is High Time for a Paradigm Shift. Springer, pp 471–526

[15] Bennett DA, Yu L, De Jager PL (2014) Building a pipeline to discover and validate novel therapeutic targets and lead compounds for Alzheimer’s disease. Biochem Pharmacol 88:617–630 24508835 10.1016/j.bcp.2014.01.037 PMC4054869

[16] Better MA (2023) Alzheimer’s disease facts and figures. In: Alzheimers Dement. pp 1598–1695 10.1002/alz.13016 36918389

[17] Boros BD, Greathouse KM, Gentry EG, Curtis KA, Birchall EL, Gearing M, Herskowitz JH (2017) Dendritic spines provide cognitive resilience against A lzheimer’s disease. Ann Neurol 82:602–614 28921611 10.1002/ana.25049 PMC5744899

[18] Botella Lucena P, Heneka MT (2024) Inflammatory aspects of Alzheimer’s disease. Acta Neuropathol 148:31 39196440 10.1007/s00401-024-02790-2

[19] Braak H, Braak E (1991) Neuropathological stageing of Alzheimer-related changes. Acta Neuropathol 82:239–259 1759558 10.1007/BF00308809

[20] Bradburn S, Murgatroyd C, Ray N (2019) Neuroinflammation in mild cognitive impairment and Alzheimer’s disease: A meta-analysis. Ageing Res Rev 50:1–8 30610927 10.1016/j.arr.2019.01.002

[21] Brunden KR, Zhang B, Carroll J, Yao Y, Potuzak JS, Hogan A-ML, Iba M, James MJ, Xie SX, Ballatore C (2010) Epothilone D improves microtubule density, axonal integrity, and cognition in a transgenic mouse model of tauopathy. J Neurosci 30:13861–13866 20943926 10.1523/JNEUROSCI.3059-10.2010 PMC2958430

[22] Bucci M, Chiotis K, Nordberg A, Initiative AsDN (2021) Alzheimer’s disease profiled by fluid and imaging markers: tau PET best predicts cognitive decline. Mol Psychiatry 26:5888–5898 34593971 10.1038/s41380-021-01263-2 PMC8758489

[23] Buendía D, Guncay T, Oyanedel M, Lemus M, Weinstein A, Ardiles ÁO, Marcos J, Fernandes A, Zângaro R, Muñoz P (2022) The transcranial light therapy improves synaptic plasticity in the Alzheimer’s disease mouse model. Brain Sci 12:1272 36291206 10.3390/brainsci12101272 PMC9599908

[24] Chen J, Long Z, Li Y, Luo M, Luo S, He G (2019) Alteration of the Wnt/GSK3β/β-catenin signalling pathway by rapamycin ameliorates pathology in an Alzheimer’s disease model. Int J Mol Med 44:313–323 31115485 10.3892/ijmm.2019.4198

[25] Chen Y, Yu Y (2023) Tau and neuroinflammation in Alzheimer’s disease: interplay mechanisms and clinical translation. J Neuroinflammation 20:165 37452321 10.1186/s12974-023-02853-3 PMC10349496

[26] Chowdhury S (2023) Monoclonal antibody treatments for Alzheimer’s disease: aducanumab and lecanemab. Discoveries Basel 11:e173 10.15190/d.2023.12 PMC11671221 39726673

[27] Clark CM, Pontecorvo MJ, Beach TG, Bedell BJ, Coleman RE, Doraiswamy PM, Fleisher AS, Reiman EM, Sabbagh MN, Sadowsky CH (2012) Cerebral PET with florbetapir compared with neuropathology at autopsy for detection of neuritic amyloid-β plaques: a prospective cohort study. Lancet Neurol 11:669–678 22749065 10.1016/S1474-4422(12)70142-4

[28] Cogswell PM, Andrews TJ, Barakos JA, Barkhof F, Bash S, Benayoun MD, Chiang GC, Franceschi AM, Jack CR, Pillai JJ (2025) Alzheimer disease anti-amyloid immunotherapies: imaging recommendations and practice considerations for monitoring of amyloid-related imaging abnormalities. AJNR Am J Neuroradiol 46:24–32 39179297 10.3174/ajnr.A8469 PMC11735439

[29] Cogswell PM, Burkett BJ, Johnson DR, Pillai JJ (2025) Altered clearance and amyloid-related imaging abnormalities. Neuroimaging Clin N Am 35:267–275 40210382 10.1016/j.nic.2024.12.002

[30] Coimbra JR, Resende R, Custódio JB, Salvador JA, Santos AE (2024) BACE1 inhibitors for Alzheimer’s disease: current challenges and future perspectives. J Alzheimers Dis 101:S53–S78 38943390 10.3233/JAD-240146

[31] Congdon EE, Ji C, Tetlow AM, Jiang Y, Sigurdsson EM (2023) Tau-targeting therapies for Alzheimer disease: current status and future directions. Nat Rev Neurol 19:715–736 37875627 10.1038/s41582-023-00883-2 PMC10965012

[32] Crespi GA, Hermans SJ, Parker MW, Miles LA (2015) Molecular basis for mid-region amyloid-β capture by leading Alzheimer’s disease immunotherapies. Sci Rep 5:9649 25880481 10.1038/srep09649 PMC4549621

[33] Cummings J (2023) Anti-amyloid monoclonal antibodies are transformative treatments that redefine Alzheimer’s disease therapeutics. Drugs 83:569–576 37060386 10.1007/s40265-023-01858-9 PMC10195708

[34] Cummings J, Osse AML, Cammann D, Powell J, Chen J (2023) Anti-amyloid monoclonal antibodies for the treatment of Alzheimer’s disease. BioDrugs 38:5–22 37955845 10.1007/s40259-023-00633-2 PMC10789674

[35] Cummings J, Zhou Y, Lee G, Zhong K, Fonseca J, Cheng F (2024) Alzheimer’s disease drug development pipeline: 2024. Alzheimers Dement Transl Res Clin Interv 10:e12465 10.1002/trc2.12465 PMC11040692 38659717

[36] Cummings JL, Osse AML, Kinney JW (2023) Alzheimer’s disease: novel targets and investigational drugs for disease modification. Drugs 83:1387–1408 37728864 10.1007/s40265-023-01938-w PMC10582128

[37] Cummings JL, Zhou Y, Van Stone A, Cammann D, Tonegawa-Kuji R, Fonseca J, Cheng F (2025) Drug repurposing for Alzheimer’s disease and other neurodegenerative disorders. Nat Commun 16:1755 39971900 10.1038/s41467-025-56690-4 PMC11840136

[38] De Strooper B, Karran E (2024) New precision medicine avenues to the prevention of Alzheimer’s disease from insights into the structure and function of γ-secretases. EMBO J 43:887–903 38396302 10.1038/s44318-024-00057-w PMC10943082

[39] Deng Q, Wu C, Parker E, Liu TC-Y, Duan R, Yang L (2024) Microglia and astrocytes in Alzheimer’s disease: significance and summary of recent advances. Aging Dis 15:1537 37815901 10.14336/AD.2023.0907 PMC11272214

[40] Dodel R, Frölich L (2025) Donanemab for Alzheimer’s disease: from preclinical research to the clinical application. Expert Rev Neurother 25:1151–1163 40790925 10.1080/14737175.2025.2546868

[41] Doran SJ, Sawyer RP (2024) Risk factors in developing amyloid related imaging abnormalities (ARIA) and clinical implications. Front Neurosci 18:1326784 38312931 10.3389/fnins.2024.1326784 PMC10834650

[42] Dubois B, von Arnim CA, Burnie N, Bozeat S, Cummings J (2023) Biomarkers in Alzheimer’s disease: role in early and differential diagnosis and recognition of atypical variants. Alzheimers Res Ther 15:175 37833762 10.1186/s13195-023-01314-6 PMC10571241

[43] Ebell MH, Barry HC, Baduni K, Grasso G (2024) Clinically important benefits and harms of monoclonal antibodies targeting amyloid for the treatment of Alzheimer disease: a systematic review and meta-analysis. Ann Fam Med 22:50–62 38253509 10.1370/afm.3050 PMC11233076

[44] Echeverria V, Barreto EG, Ávila-Rodriguezc M, Tarasov VV, Aliev G (2017) Is VEGF a key target of cotinine and other potential therapies against Alzheimer disease? Curr Alzheimer Res 14:1155–1163 28356047 10.2174/1567205014666170329113007

[45] Fedele E (2023) Anti-amyloid therapies for Alzheimer’s disease and the amyloid cascade hypothesis. Int J Mol Sci 24:14499 37833948 10.3390/ijms241914499 PMC10578107

[46] Filippi M, Cecchetti G, Spinelli EG, Vezzulli P, Falini A, Agosta F (2022) Amyloid-related imaging abnormalities and β-amyloid–targeting antibodies: a systematic review. JAMA Neurol 79:291–304 35099507 10.1001/jamaneurol.2021.5205

[47] Forgrave LM, Ma M, Best JR, DeMarco ML (2019) The diagnostic performance of neurofilament light chain in CSF and blood for Alzheimer’s disease, frontotemporal dementia, and amyotrophic lateral sclerosis: a systematic review and meta-analysis. Alzheimers Dement (Amst) 11:730–743 31909174 10.1016/j.dadm.2019.08.009 PMC6939029

[48] Frost GR, Li Y-M (2017) The role of astrocytes in amyloid production and Alzheimer’s disease. Open Biol 7:170228 29237809 10.1098/rsob.170228 PMC5746550

[49] Gao C, Jiang J, Tan Y, Chen S (2023) Microglia in neurodegenerative diseases: mechanism and potential therapeutic targets. Signal Transduct Target Ther 8:359 37735487 10.1038/s41392-023-01588-0 PMC10514343

[50] Gao N, Xing F, Du J, Zhao W, Wang S, Chen M, Dong X, Qi Y (2025) The manipulator behind" scissors": γ-Secretase and its modulators in Alzheimer’s disease. Front Aging Neurosci 17:1637671 40927393 10.3389/fnagi.2025.1637671 PMC12414960

[51] Gertsik N, Chiu D, Li Y-M (2015) Complex regulation of γ-secretase: from obligatory to modulatory subunits. Front Aging Neurosci 6:342 25610395 10.3389/fnagi.2014.00342 PMC4285130

[52] Gildea HK, Liddelow SA (2025) Mechanisms of astrocyte aging in reactivity and disease. Mol Neurodegener 20:1–14 39979986 10.1186/s13024-025-00810-7 PMC11844071

[53] Goldman JS (2012) New approaches to genetic counseling and testing for Alzheimer’s disease and frontotemporal degeneration. Curr Neurol Neurosci Rep 12:502–510 22773362 10.1007/s11910-012-0296-1 PMC3437002

[54] Graff-Radford J, Yong KX, Apostolova LG, Bouwman FH, Carrillo M, Dickerson BC, Rabinovici GD, Schott JM, Jones DT, Murray ME (2021) New insights into atypical Alzheimer’s disease in the era of biomarkers. Lancet Neurol 20:222–234 33609479 10.1016/S1474-4422(20)30440-3 PMC8056394

[55] Greenberg SM, Bax F, van Veluw SJ (2025) Amyloid-related imaging abnormalities: manifestations, metrics and mechanisms. Nat Rev Neurol 21:193–203 39794509 10.1038/s41582-024-01053-8

[56] Grimm HP, Schumacher V, Schäfer M, Imhof-Jung S, Freskgård P-O, Brady K, Hofmann C, Rüger P, Schlothauer T, Göpfert U (2023) Delivery of the Brainshuttle™ amyloid-beta antibody fusion trontinemab to non-human primate brain and projected efficacious dose regimens in humans. In: MAbs. Taylor & Francis, p 2261509 10.1080/19420862.2023.2261509 PMC10572082 37823690

[57] Grothe MJ, Moscoso A, Ashton NJ, Karikari TK, Lantero-Rodriguez J, Snellman A, Zetterberg H, Blennow K, Schöll M, Initiative AsDN (2021) Associations of fully automated CSF and novel plasma biomarkers with Alzheimer disease neuropathology at autopsy. Neurology 97:e1229–e1242 34266917 10.1212/WNL.0000000000012513 PMC8480485

[58] Gunes S, Aizawa Y, Sugashi T, Sugimoto M, Rodrigues PP (2022) Biomarkers for Alzheimer’s disease in the current state: a narrative review. Int J Mol Sci 23:4962 35563350 10.3390/ijms23094962 PMC9102515

[59] Hampel H, Cummings J, Blennow K, Gao P, Jack CR Jr, Vergallo A (2021) Developing the ATX (N) classification for use across the Alzheimer disease continuum. Nat Rev Neurol 17:580–589 34239130 10.1038/s41582-021-00520-w

[60] Hampel H, Elhage A, Cho M, Apostolova LG, Nicoll JA, Atri A (2023) Amyloid-related imaging abnormalities (ARIA): radiological, biological and clinical characteristics. Brain 146:4414–4424 37280110 10.1093/brain/awad188 PMC10629981

[61] Hampel H, Hardy J, Blennow K, Chen C, Perry G, Kim SH, Villemagne VL, Aisen P, Vendruscolo M, Iwatsubo T (2021) The amyloid-β pathway in Alzheimer’s disease. Mol Psychiatry 26:5481–5503 34456336 10.1038/s41380-021-01249-0 PMC8758495

[62] Hampel H, Hu Y, Cummings J, Mattke S, Iwatsubo T, Nakamura A, Vellas B, O’Bryant S, Shaw LM, Cho M (2023) Blood-based biomarkers for Alzheimer’s disease: current state and future use in a transformed global healthcare landscape. Neuron 111:2781–2799 37295421 10.1016/j.neuron.2023.05.017 PMC10720399

[63] Hansson O, Blennow K, Zetterberg H, Dage J (2023) Blood biomarkers for Alzheimer’s disease in clinical practice and trials. Nat Aging 3:506–519 37202517 10.1038/s43587-023-00403-3 PMC10979350

[64] Hardy JA, Higgins GA (1992) Alzheimer’s disease: the amyloid cascade hypothesis. Science 256:184–185 1566067 10.1126/science.1566067

[65] Heneka MT, Carson MJ, El Khoury J, Landreth GE, Brosseron F, Feinstein DL, Jacobs AH, Wyss-Coray T, Vitorica J, Ransohoff RM (2015) Neuroinflammation in Alzheimer’s disease. Lancet Neurol 14:388–405 25792098 10.1016/S1474-4422(15)70016-5 PMC5909703

[66] Heneka MT, Kummer MP, Stutz A, Delekate A, Schwartz S, Vieira-Saecker A, Griep A, Axt D, Remus A, Tzeng T-C (2013) NLRP3 is activated in Alzheimer’s disease and contributes to pathology in APP/PS1 mice. Nature 493:674–678 23254930 10.1038/nature11729 PMC3812809

[67] Herrup K, Carrillo MC, Schenk D, Cacace A, DeSanti S, Fremeau R, Bhat R, Glicksman M, May P, Swerdlow R (2013) Beyond amyloid: getting real about nonamyloid targets in Alzheimer’s disease. Alzheimers Dement 9(452–458):e451 10.1016/j.jalz.2013.01.017 PMC3983562 23809366

[68] Hu X, Fan Q, Hou H, Yan R (2016) Neurological dysfunctions associated with altered BACE 1-dependent Neuregulin-1 signaling. J Neurochem 136:234–249 26465092 10.1111/jnc.13395 PMC4833723

[69] Husna IN, Yahaya MF, Mohamed W, Teoh SL, Hui CK, Kumar J (2020) Pharmacotherapy of Alzheimer’s disease: seeking clarity in a time of uncertainty. Front Pharmacol 11:261 32265696 10.3389/fphar.2020.00261 PMC7105678

[70] Iqbal K, Liu F, Gong C-X (2016) Tau and neurodegenerative disease: the story so far. Nat Rev Neurol 12:15–27 26635213 10.1038/nrneurol.2015.225

[71] Italiani P, Puxeddu I, Napoletano S, Scala E, Melillo D, Manocchio S, Angiolillo A, Migliorini P, Boraschi D, Vitale E (2018) Circulating levels of IL-1 family cytokines and receptors in Alzheimer’s disease: new markers of disease progression? J Neuroinflammation 15:342 30541566 10.1186/s12974-018-1376-1 PMC6292179

[72] Jang K, Garraway SM (2025) TrkB agonist (7, 8-DHF)-induced responses in dorsal root ganglia neurons are decreased after spinal cord injury: implication for peripheral pain mechanisms. eNeuro. 10.1523/eneuro.0219-24.2024 39753357 10.1523/ENEURO.0219-24.2024 PMC11728855

[73] Jayadev S, Leverenz JB, Steinbart E, Stahl J, Klunk W, Yu C-E, Bird TD (2010) Alzheimer’s disease phenotypes and genotypes associated with mutations in presenilin 2. Brain 133:1143–1154 20375137 10.1093/brain/awq033 PMC2850581

[74] Jayaprakash N, Elumalai K (2025) Translational medicine in Alzheimer’s disease: the journey of Donanemab from discovery to clinical application. Chronic Dis Transl Med 11:105–116 40486952 10.1002/cdt3.155 PMC12142702

[75] Johannesson M, Söderberg L, Zachrisson O, Fritz N, Kylefjord H, Gkanatsiou E, Button E, Svensson A-S, Rachalski A, Nygren P (2024) Lecanemab demonstrates highly selective binding to Aβ protofibrils isolated from Alzheimer’s disease brains. Mol Cell Neurosci 130:103949 38906341 10.1016/j.mcn.2024.103949

[76] Kamatham PT, Shukla R, Khatri DK, Vora LK (2024) Pathogenesis, diagnostics, and therapeutics for Alzheimer’s disease: breaking the memory barrier. Ageing Res Rev 101:102481 39236855 10.1016/j.arr.2024.102481

[77] Kang JH, Korecka M, Lee EB, Cousins KA, Tropea TF, Chen-Plotkin AA, Irwin DJ, Wolk D, Brylska M, Wan Y (2023) Alzheimer disease biomarkers: moving from CSF to plasma for reliable detection of amyloid and tau pathology. Clin Chem 69:1247–1259 37725909 10.1093/clinchem/hvad139 PMC10895336

[78] Karch CM, Cruchaga C, Goate AM (2014) Alzheimer’s disease genetics: from the bench to the clinic. Neuron 83:11–26 24991952 10.1016/j.neuron.2014.05.041 PMC4120741

[79] Keren-Shaul H, Spinrad A, Weiner A, Matcovitch-Natan O, Dvir-Szternfeld R, Ulland TK, David E, Baruch K, Lara-Astaiso D, Toth B (2017) A unique microglia type associated with restricting development of Alzheimer’s disease. Cell 169(1276–1290):e1217 10.1016/j.cell.2017.05.018 28602351

[80] Khachaturian Z (2025) History of Alzheimer’s disease research centers: from inception in 1984 to evolution beyond 2025. Alzheimers Dement 21:e70778 41117350 10.1002/alz.70778 PMC12538636

[81] Khalaf SS, Hafez MM, Mehanna ET, Mesbah NM, Abo-Elmatty DM (2019) Combined vildagliptin and memantine treatment downregulates expression of amyloid precursor protein, and total and phosphorylated tau in a rat model of combined Alzheimer’s disease and type 2 diabetes. Naunyn Schmiedebergs Arch Pharmacol 392:685–695 30759264 10.1007/s00210-019-01616-3

[82] Kim AY, Al Jerdi S, MacDonald R, Triggle CR (2024) Alzheimer’s disease and its treatment–yesterday, today, and tomorrow. Front Pharmacol 15:1399121 38868666 10.3389/fphar.2024.1399121 PMC11167451

[83] Kim SY, Jin CY, Kim CH, Yoo YH, Choi SH, Kim GY, Yoon HM, Park HT, Choi YH (2019) Isorhamnetin alleviates lipopolysaccharide-induced inflammatory responses in BV2 microglia by inactivating NF-κB, blocking the TLR4 pathway and reducing ROS generation. Int J Mol Med 43:682–692 30483725 10.3892/ijmm.2018.3993 PMC6317673

[84] Klyucherev TO, Olszewski P, Shalimova AA, Chubarev VN, Tarasov VV, Attwood MM, Syvänen S, Schiöth HB (2022) Advances in the development of new biomarkers for Alzheimer’s disease. Translat Neurodegen 11:25 10.1186/s40035-022-00296-z PMC9027827 35449079

[85] Koelsch G (2017) BACE1 function and inhibition: implications of intervention in the amyloid pathway of Alzheimer’s disease pathology. Molecules 22:1723 29027981 10.3390/molecules22101723 PMC6151801

[86] Krasemann S, Madore C, Cialic R, Baufeld C, Calcagno N, El Fatimy R, Beckers L, O’loughlin E, Xu Y, Fanek Z (2017) The TREM2-APOE pathway drives the transcriptional phenotype of dysfunctional microglia in neurodegenerative diseases. Immunity 47(3):566–581 28930663 10.1016/j.immuni.2017.08.008 PMC5719893

[87] Lawrence JM, Schardien K, Wigdahl B, Nonnemacher MR (2023) Roles of neuropathology-associated reactive astrocytes: a systematic review. Acta Neuropathol Commun 11:42 36915214 10.1186/s40478-023-01526-9 PMC10009953

[88] Leng F, Edison P (2021) Neuroinflammation and microglial activation in Alzheimer disease: where do we go from here? Nat Rev Neurol 17:157–172 33318676 10.1038/s41582-020-00435-y

[89] Leuzy A, Mattsson-Carlgren N, Palmqvist S, Janelidze S, Dage JL, Hansson O (2022) Blood-based biomarkers for Alzheimer’s disease. EMBO Mol Med 14:e14408 34859598 10.15252/emmm.202114408 PMC8749476

[90] Li T, Martin E, Abada Y-s, Boucher C, Cès A, Youssef I, Fenaux G, Forand Y, Legrand A, Nachiket N (2020) Effects of chronic masitinib treatment in APPswe/PSEN1dE9 transgenic mice modeling Alzheimer’s disease. J Alzheimer’s Disease 76:1339–1345 32623401 10.3233/JAD-200466

[91] Liddelow SA, Guttenplan KA, Clarke LE, Bennett FC, Bohlen CJ, Schirmer L, Bennett ML, Münch AE, Chung W-S, Peterson TC (2017) Neurotoxic reactive astrocytes are induced by activated microglia. Nature 541:481–487 28099414 10.1038/nature21029 PMC5404890

[92] Lista S, Imbimbo BP, Grasso M, Fidilio A, Emanuele E, Minoretti P, López-Ortiz S, Martín-Hernández J, Gabelle A, Caruso G (2024) Tracking neuroinflammatory biomarkers in Alzheimer’s disease: a strategy for individualized therapeutic approaches? J Neuroinflammation 21:187 39080712 10.1186/s12974-024-03163-y PMC11289964

[93] Liu L, Ding L, Rovere M, Wolfe MS, Selkoe DJ (2019) A cellular complex of BACE1 and γ-secretase sequentially generates Aβ from its full-length precursor. J Cell Biol 218:644–663 30626721 10.1083/jcb.201806205 PMC6363461

[94] Livingston G, Huntley J, Sommerlad A, Ames D, Ballard C, Banerjee S, Brayne C, Burns A, Cohen-Mansfield J, Cooper C (2020) Dementia prevention, intervention, and care: 2020 report of the Lancet Commission. The lancet 396:413–446 10.1016/S0140-6736(20)30367-6 PMC7392084 32738937

[95] Long JM, Holtzman DM (2019) Alzheimer disease: an update on pathobiology and treatment strategies. Cell 179:312–339 31564456 10.1016/j.cell.2019.09.001 PMC6778042

[96] Mahaman YAR, Embaye KS, Huang F, Li L, Zhu F, Wang J-Z, Liu R, Feng J, Wang X (2022) Biomarkers used in Alzheimer’s disease diagnosis, treatment, and prevention. Ageing Res Rev 74:101544 34933129 10.1016/j.arr.2021.101544

[97] Mathew J, Maroky AS, Sinduraj S, Chandrababu A (2025) Integrative systems biology and multi-omics approaches in Alzheimer’s disease: briding biomarkers, neuroinflammation, and precision medicine. Int J Appl Pharmac 17:107–121

[98] Mawuenyega KG, Sigurdson W, Ovod V, Munsell L, Kasten T, Morris JC, Yarasheski KE, Bateman RJ (2010) Decreased clearance of CNS β-amyloid in Alzheimer’s disease. Science 330:1774–1774 21148344 10.1126/science.1197623 PMC3073454

[99] McDade E, Wang G, Gordon BA, Hassenstab J, Benzinger TL, Buckles V, Fagan AM, Holtzman DM, Cairns NJ, Goate AM (2018) Longitudinal cognitive and biomarker changes in dominantly inherited Alzheimer disease. Neurology 91:e1295–e1306 30217935 10.1212/WNL.0000000000006277 PMC6177272

[100] Mecca AP, Chen MK, O’Dell RS, Naganawa M, Toyonaga T, Godek TA, Harris JE, Bartlett HH, Zhao W, Nabulsi NB (2020) In vivo measurement of widespread synaptic loss in Alzheimer’s disease with SV2A PET. Alzheimers Dement 16:974–982 32400950 10.1002/alz.12097 PMC7383876

[101] Meglio M (2023) Eli Lilly's Remternetug Demonstrates Significant Amyloid Plaque Removal in Early-Stage Trial. Neurology Live:NA-NA

[102] Minoshima S, Cross D, Thientunyakit T, Foster NL, Drzezga A (2022) 18F-FDG PET imaging in neurodegenerative dementing disorders: insights into subtype classification, emerging disease categories, and mixed dementia with copathologies. J Nucl Med 63:2S-12S 35649653 10.2967/jnumed.121.263194

[103] Mintun MA, Lo AC, Duggan Evans C, Wessels AM, Ardayfio PA, Andersen SW, Shcherbinin S, Sparks J, Sims JR, Brys M (2021) Donanemab in early Alzheimer’s disease. N Engl J Med 384:1691–1704 33720637 10.1056/NEJMoa2100708

[104] Moirangthem R, Bar-On Y (2025) Passive immunization in the prevention and treatment of viral infections. Eur J Immunol 55:e202451606 40415212 10.1002/eji.202451606 PMC12104555

[105] Nam Y, Moon GJ, Kim SR (2021) Therapeutic potential of AAV1-Rheb (S16H) transduction against neurodegenerative diseases. Int J Mol Sci 22:3064 33802760 10.3390/ijms22063064 PMC8002454

[106] Narasimhan S, Holtzman DM, Apostolova LG, Cruchaga C, Masters CL, Hardy J, Villemagne VL, Bell J, Cho M, Hampel H (2024) Apolipoprotein E in Alzheimer’s disease trajectories and the next-generation clinical care pathway. Nat Neurosci 27:1236–1252 38898183 10.1038/s41593-024-01669-5

[107] Nasrolahi A, Javaherforooshzadeh F, Jafarzadeh-Gharehziaaddin M, Mahmoudi J, Asl KD, Shabani Z (2022) Therapeutic potential of neurotrophic factors in Alzheimer’s disease. Mol Biol Rep 49:2345–2357 34826049 10.1007/s11033-021-06968-9

[108] Neumann H, Kotter MR, Franklin RJ (2009) Debris clearance by microglia: an essential link between degeneration and regeneration. Brain 132:288–295 18567623 10.1093/brain/awn109 PMC2640215

[109] Nijs J, Malfliet A, Nishigami T (2023) Nociplastic pain and central sensitization in patients with chronic pain conditions: a terminology update for clinicians. Braz J Phys Ther 27:100518 37348359 10.1016/j.bjpt.2023.100518 PMC10314229

[110] Nosheny RL, Yen D, Howell T, Camacho M, Moulder K, Gummadi S, Bui C, Kannan S, Ashford MT, Knight K (2023) Evaluation of the electronic clinical dementia rating for dementia screening. JAMA Netw Open 6:e2333786–e2333786 37707812 10.1001/jamanetworkopen.2023.33786 PMC10502518

[111] Ohno M (2025) BACE1 as an early biomarker and its relevance to risk factors for Alzheimer’s disease. Brain Res Bull. 10.1016/j.brainresbull.2025.111475 40706761 10.1016/j.brainresbull.2025.111475

[112] Ohno M (2025) BACE1 at the crossroads of a vicious circle between Alzheimer’s disease and diabetes mellitus. Front Dementia 4:1730524 10.3389/frdem.2025.1730524 PMC12722518 41446639

[113] Olmos-Alonso A, Schetters ST, Sri S, Askew K, Mancuso R, Vargas-Caballero M, Holscher C, Perry VH, Gomez-Nicola D (2016) Pharmacological targeting of CSF1R inhibits microglial proliferation and prevents the progression of Alzheimer’s-like pathology. Brain 139:891–907 26747862 10.1093/brain/awv379 PMC4766375

[114] Orciani C, Hall H, Pentz R, Foret MK, Do Carmo S, Cuello AC (2022) Long-term nucleus basalis cholinergic depletion induces attentional deficits and impacts cortical neurons and BDNF levels without affecting the NGF synthesis. J Neurochem 163:149–167 35921478 10.1111/jnc.15683

[115] Ossenkoppele R, Pichet Binette A, Groot C, Smith R, Strandberg O, Palmqvist S, Stomrud E, Tideman P, Ohlsson T, Jögi J (2022) Amyloid and tau PET-positive cognitively unimpaired individuals are at high risk for future cognitive decline. Nat Med 28:2381–2387 36357681 10.1038/s41591-022-02049-x PMC9671808

[116] Owens DK, Davidson KW, Krist AH, Barry MJ, Cabana M, Caughey AB, Doubeni CA, Epling JW, Kubik M, Landefeld CS (2020) Screening for cognitive impairment in older adults: US Preventive Services Task Force recommendation statement. JAMA 323:757–763 32096858 10.1001/jama.2020.0435

[117] Pan X-d, Zhu Y-g, Lin N, Zhang J, Ye Q-y, Huang H-p, Chen X-c (2011) Microglial phagocytosis induced by fibrillar β-amyloid is attenuated by oligomeric β-amyloid: implications for Alzheimer’s disease. Mol Neurodegener 6:45 21718498 10.1186/1750-1326-6-45 PMC3149591

[118] Papadopoulou MA, Rogdakis T, Charou D, Peteinareli M, Ntarntani K, Gravanis A, Chanoumidou K, Charalampopoulos I (2023) Neurotrophin analog ENT-A044 activates the p75 neurotrophin receptor, regulating neuronal survival in a cell context-dependent manner. Int J Mol Sci 24:11683 37511441 10.3390/ijms241411683 PMC10380564

[119] Perneczky R, Dom G, Chan A, Falkai P, Bassetti C (2024) Anti-amyloid antibody treatments for Alzheimer’s disease. Eur J Neurol 31:e16049 37697714 10.1111/ene.16049 PMC11235913

[120] Poniah P, Abdul Rashed A, Abdul Jalil J, Ali EZ (2025) Clinical significance of early-onset Alzheimer’s mutations in Asian and Western populations: a scoping review. Genes Basel 16:345 40149496 10.3390/genes16030345 PMC11942072

[121] Pratsch K, Unemura C, Ito M, Lichtenthaler SF, Horiguchi N, Herms J (2023) New highly selective BACE1 inhibitors and their effects on dendritic spine density in vivo. Int J Mol Sci 24:12283 37569661 10.3390/ijms241512283 PMC10418759

[122] Qi S, Yu J, Li L, Dong C, Ji Z, Cao L, Wei Z, Liang Z (2024) Advances in non-invasive brain stimulation: enhancing sports performance function and insights into exercise science. Front Hum Neurosci 18:1477111 39677404 10.3389/fnhum.2024.1477111 PMC11638246

[123] Rabinovici GD, La Joie R (2023) Amyloid-targeting monoclonal antibodies for Alzheimer disease. JAMA 330:507–509 37459124 10.1001/jama.2023.11703

[124] Rafii MS, Aisen PS (2025) Amyloid-lowering immunotherapies for Alzheimer disease: current status and future directions. Nat Rev Neurol 21:490–498 40691719 10.1038/s41582-025-01123-5

[125] Ribaldi F, Altomare D, Frisoni G (2019) Is a large-scale screening for Alzheimer’s disease possible? Yes, in a few years. J Prev Alzheimers Dis 6:221–222 31686091 10.14283/jpad.2019.29

[126] Scheltens P, De Strooper B, Kivipelto M, Holstege H, Chételat G, Teunissen CE, Cummings J, van der Flier WM (2021) Alzheimer’s disease. Lancet 397:1577–1590 33667416 10.1016/S0140-6736(20)32205-4 PMC8354300

[127] Selles MC, Fortuna JT, Zappa-Villar MF, de Faria YP, Souza AS, Suemoto CK, Leite RE, Rodriguez RD, Grinberg LT, Reggiani PC (2020) Adenovirus-mediated transduction of insulin-like growth factor 1 protects hippocampal neurons from the toxicity of Aβ oligomers and prevents memory loss in an Alzheimer mouse model. Mol Neurobiol 57:1473–1483 31760608 10.1007/s12035-019-01827-y PMC7412754

[128] Simpson DS, Oliver PL (2020) ROS generation in microglia: understanding oxidative stress and inflammation in neurodegenerative disease. Antioxidants Basel 9:743 32823544 10.3390/antiox9080743 PMC7463655

[129] Sims JR, Zimmer JA, Evans CD, Lu M, Ardayfio P, Sparks J, Wessels AM, Shcherbinin S, Wang H, Nery ESM (2023) Donanemab in early symptomatic Alzheimer disease: the TRAILBLAZER-ALZ 2 randomized clinical trial. JAMA 330:512–527 37459141 10.1001/jama.2023.13239 PMC10352931

[130] Smith JA, Das A, Ray SK, Banik NL (2012) Role of pro-inflammatory cytokines released from microglia in neurodegenerative diseases. Brain Res Bull 87:10–20 22024597 10.1016/j.brainresbull.2011.10.004 PMC9827422

[131] Swanson CJ, Zhang Y, Dhadda S, Wang J, Kaplow J, Lai RY, Lannfelt L, Bradley H, Rabe M, Koyama A (2021) A randomized, double-blind, phase 2b proof-of-concept clinical trial in early Alzheimer’s disease with Lecanemab, an anti-Aβ protofibril antibody. Alzheimers Res Ther 13:80 33865446 10.1186/s13195-021-00813-8 PMC8053280

[132] Tate B, McKee TD, Loureiro RM, Dumin JA, Xia W, Pojasek K, Austin WF, Fuller NO, Hubbs JL, Shen R (2012) Modulation of gamma-secretase for the treatment of Alzheimer’s disease. Int J Alzheimers Dis 2012:210756 23320246 10.1155/2012/210756 PMC3536039

[133] Tönnies E, Trushina E (2017) Oxidative stress, synaptic dysfunction, and Alzheimer’s disease. J Alzheimers Dis 57:1105–1121 28059794 10.3233/JAD-161088 PMC5409043

[134] Ullah R, Lee EJ (2023) Advances in amyloid-β clearance in the brain and periphery: implications for neurodegenerative diseases. Exp Neurobiol 32:216 37749925 10.5607/en23014 PMC10569141

[135] Ulland TK, Colonna M (2018) TREM2—a key player in microglial biology and Alzheimer disease. Nat Rev Neurol 14:667–675 30266932 10.1038/s41582-018-0072-1

[136] Van Dyck CH, Swanson CJ, Aisen P, Bateman RJ, Chen C, Gee M, Kanekiyo M, Li D, Reyderman L, Cohen S (2023) Lecanemab in early Alzheimer’s disease. N Engl J Med 388:9–21 36449413 10.1056/NEJMoa2212948

[137] Varesi A, Carrara A, Pires VG, Floris V, Pierella E, Savioli G, Prasad S, Esposito C, Ricevuti G, Chirumbolo S (2022) Blood-based biomarkers for Alzheimer’s disease diagnosis and progression: an overview. Cells 11:1367 35456047 10.3390/cells11081367 PMC9044750

[138] Vishnumukkala T, Che Mohd Nassir CMN, Hein ZM, Kalerammana Gopalakrishna P, Karikalan B, Alkatiri A, Jagadeesan S, Naik VR, Thomas W, Mohd Moklas MA (2025) Glial Cells as Emerging Therapeutic Targets in Neurodegenerative Diseases: Mechanistic Insights and Translational Perspectives. Cells 14:1497 41090725 10.3390/cells14191497 PMC12523653

[139] Volloch V, Rits-Volloch S (2025) Production of Amyloid-β in the Aβ-Protein-Precursor Proteolytic Pathway Is Discontinued or Severely Suppressed in Alzheimer’s Disease-Affected Neurons: Contesting the ‘Obvious.’ Genes 16:46 39858593 10.3390/genes16010046 PMC11764795

[140] Wang Q, Chen S, Wang J, Shang H, Chen X (2024) Advancements in pharmacological treatment of Alzheimer’s disease: the advent of disease-modifying therapies (DMTs). Brain Sci 14:990 39452004 10.3390/brainsci14100990 PMC11506318

[141] Watkins EA, Vassar R (2024) BACE inhibitor clinical trials for Alzheimer’s disease. J Alzheimers Dis 101:S41–S52 39422943 10.3233/JAD-231258

[142] Wattmo C, Blennow K, Hansson O (2020) Cerebro-spinal fluid biomarker levels: phosphorylated tau (T) and total tau (N) as markers for rate of progression in Alzheimer’s disease. BMC Neurol 20:10 31918679 10.1186/s12883-019-1591-0 PMC6951013

[143] Wright R (2021) Microglia set the pace for tau spread. Nat Neurosci 24:1342–1342 34588697 10.1038/s41593-021-00931-4

[144] Wu W, Ji Y, Wang Z, Wu X, Li J, Gu F, Chen Z, Wang Z (2023) The FDA-approved anti-amyloid-β monoclonal antibodies for the treatment of Alzheimer’s disease: a systematic review and meta-analysis of randomized controlled trials. Eur J Med Res 28:544 38017568 10.1186/s40001-023-01512-w PMC10683264

[145] Zhang J, Zhang Y, Wang J, Xia Y, Zhang J, Chen L (2024) Recent advances in Alzheimer’s disease: mechanisms, clinical trials and new drug development strategies. Signal Transduct Target Ther 9:211 39174535 10.1038/s41392-024-01911-3 PMC11344989

[146] Zhang Y, Chen H, Li R, Sterling K, Song W (2023) Amyloid β-based therapy for Alzheimer’s disease: challenges, successes and future. Signal Transduct Target Ther 8:248 37386015 10.1038/s41392-023-01484-7 PMC10310781

[147] Zhao R, Ren B, Xiao Y, Tian J, Zou Y, Wei J, Qi Y, Hu A, Xie X, Huang ZJ (2024) Axo-axonic synaptic input drives homeostatic plasticity by tuning the axon initial segment structurally and functionally. Sci Adv 10:eadk4331 39093969 10.1126/sciadv.adk4331 PMC11296346

[148] Zimmer ER, Leuzy A, Benedet AL, Breitner J, Gauthier S, Rosa-Neto P (2014) Tracking neuroinflammation in Alzheimer’s disease: the role of positron emission tomography imaging. J Neuroinflammation 11:120 25005532 10.1186/1742-2094-11-120 PMC4099095

[149] Zimmer JA, Ardayfio P, Wang H, Khanna R, Evans CD, Lu M, Sparks J, Andersen S, Lauzon S, Nery ESM (2025) Amyloid-related imaging abnormalities with donanemab in early symptomatic Alzheimer disease: secondary analysis of the TRAILBLAZER-ALZ and ALZ 2 randomized clinical trials. JAMA Neurol 82:461–469 40063015 10.1001/jamaneurol.2025.0065 PMC11894547

